# Structural basis of actin monomer re-charging by cyclase-associated protein

**DOI:** 10.1038/s41467-018-04231-7

**Published:** 2018-05-14

**Authors:** Tommi Kotila, Konstantin Kogan, Giray Enkavi, Siyang Guo, Ilpo Vattulainen, Bruce L. Goode, Pekka Lappalainen

**Affiliations:** 1 Institute of Biotechnology, University of Helsinki, 00014 Helsinki, Finland; 2 Department of Physics, University of Helsinki, 00014 Helsinki, Finland; 3 Department of Biology, Brandeis University, Waltham, MA 02453 USA; 4 Laboratory of Physics, Tampere University of Technology, 33101 Tampere, Finland

## Abstract

Actin polymerization powers key cellular processes, including motility, morphogenesis, and endocytosis. The actin turnover cycle depends critically on “re-charging” of ADP-actin monomers with ATP, but whether this reaction requires dedicated proteins in cells, and the underlying mechanism, have remained elusive. Here we report that nucleotide exchange catalyzed by the ubiquitous cytoskeletal regulator cyclase-associated protein (CAP) is critical for actin-based processes in vivo. We determine the structure of the CAP–actin complex, which reveals that nucleotide exchange occurs in a compact, sandwich-like complex formed between the dimeric actin-binding domain of CAP and two ADP-actin monomers. In the crystal structure, the C-terminal tail of CAP associates with the nucleotide-sensing region of actin, and this interaction is required for rapid re-charging of actin by both yeast and mammalian CAPs. These data uncover the conserved structural basis and biological role of protein-catalyzed re-charging of actin monomers.

## Abstract (web summary)

Depolymerized ADP-actin monomers must be recharged with ATP for new rounds of filament assembly. Here the authors show that cyclase-associated protein (CAP) catalyzes actin nucleotide exchange in vivo and their CAP–actin complex structure reveals the molecular mechanism of CAP-mediated actin nucleotide exchange.

## Introduction

The actin cytoskeleton is critical for a wide range of cellular processes, including migration, morphogenesis, and endocytosis. Consequently, defects in the regulation of actin dynamics and actin network organization are linked to a number of diseases, including cancer metastasis, immune and neurological disorders^1–3^. The rapid polymerization of actin filaments, that provides force for the above-mentioned cellular processes, must be balanced by the disassembly of “aged” actin filaments, and recycling of actin monomers for new rounds of filament assembly. This process, called “treadmilling”, consists of four phases: (1) incorporation of assembly-competent ATP-actin monomers to the rapidly growing actin filament barbed end; (2) ATP-hydrolysis, followed by Pi release, on actin subunits in the filament; (3) dissociation of ADP-actin monomers from the pointed end of filament; and (4) “re-charging” of ADP-actin monomers with ATP^4^. In vitro, these four phases, as well as nucleation of new actin filaments, are relatively slow, and thus a large collection of actin-binding proteins such as the Arp2/3 complex, formins, and ADF/cofilin, evolved to enhance the rate of actin dynamics^5–13^. While previous studies have demonstrated the in vivo importance of these proteins that catalyze actin nucleation, polymerization, and disassembly^14–18^, it has remained unclear whether additional protein machinery is also required to catalyze actin monomer “re-charging” (exchange of ADP for ATP) in cells.

Two evolutionarily conserved proteins, profilin and cyclase-associated protein (CAP), can catalyze nucleotide exchange on actin monomers in vitro. However, CAP appears to be better suited for this function, because it binds the substrate (ADP-G-actin) with much higher affinity compared to profilin^19,20^. Moreover, whereas all CAPs tested so far catalyze nucleotide exchange in vitro, only a subset of profilins accelerate nucleotide exchange in biochemical assays^21^. Finally, only CAP has been shown in vitro to effectively catalyze nucleotide exchange on cofilin-bound ADP-actin monomers^22^. These observations have called into question the popular view depicting profilin as the key driver of actin monomer recharging, and suggest instead that CAP may perform this conserved function. Until now, however, it has not been possible to rigorously test whether either or both proteins serve this function in vivo, due to an absence of mutants that disrupt nucleotide exchange activity without compromising actin binding.

CAPs are multi-domain, multifunctional proteins that oligomerize into hexamers and promote rapid actin filament turnover in vitro and in cells^22–24^. Whereas yeasts and invertebrates have only one CAP protein, vertebrates express two CAP isoforms: ubiquitously expressed CAP1 and muscle-specific CAP2^25^. The N-terminal half of CAPs binds ADF/cofilin-actin monomer complexes and actin filaments, and accelerates ADF/cofilin and twinfilin-mediated actin filament disassembly^26–30^. The C-terminal half of CAPs harbors two proline-rich regions, PP1 and PP2, which bind to profilin and SH3 domain proteins, respectively, and a Wiscott Aldrich Syndrome protein homology 2 (WH2) domain, which binds to both ADP-actin and ATP-actin monomers^20,21,31–34^. At the C-terminus of CAPs is a homo-dimeric β-sheet domain, which displays structural similarity to other, functionally unrelated proteins, including X-linked retinitis pigmentosa 2 protein (RP2), and hence is referred to as a CAP and RP2 (CARP) domain^35^. The CARP domain of CAPs binds specifically to ADP-G-actin, and together with the adjacent WH2 domain catalyzes nucleotide exchange on actin monomers^20,27,34^.

Despite the fundamental requirement of CAPs for actin cytoskeleton organization and function across the eukaryotic kingdom^36–41^, the underlying mechanism by which this protein regulates cytoskeletal dynamics in vivo has remained elusive. Moreover, the CARP domain does not display any structural homology to other known actin-binding domains, and despite extensive mutagenesis^20,42,43^ the mechanism by which CAPs associate with actin monomers and catalyze nucleotide exchange has thus remained a mystery. Here, we determined the crystal structure of CAP1/ADP-G-actin complex. Combined with molecular dynamics (MD) simulations, biochemical experiments, and in vivo studies on budding yeast, we uncover the structural basis and biological role of CAP-catalyzed nucleotide exchange on actin monomers.

## Results

### Crystal structure of CAP1_317–474_/ADP-G-actin complex

**Fig. 1:**
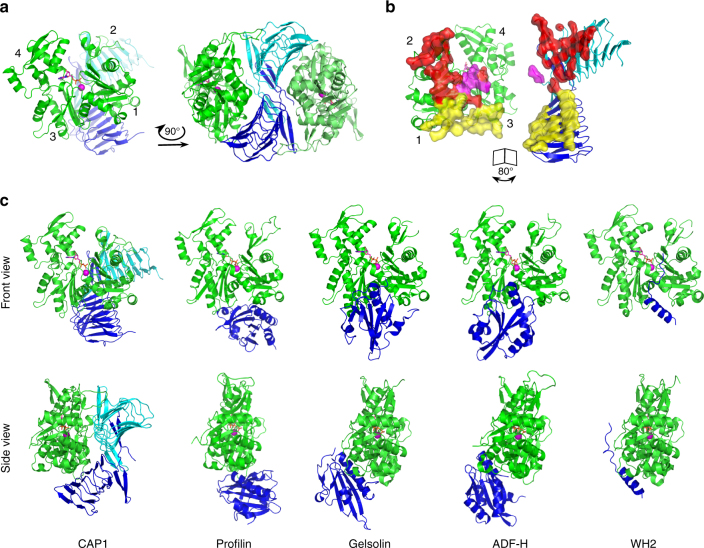
Crystal structure of the CARP domain from mouse CAP1 in complex with ADP-actin. **a** CARP domain forms a homodimer (the subunits are in blue and cyan) that binds two actin molecules (in green) through their subdomains 1, 2, and 3. **b** The CARP domain dimer covers a large surface (1944 Å^2^) on each actin monomers. The two subunits of the CARP domain dimer employ two different interfaces (primary interface in yellow; secondary interface in red; and C-terminal tail of CARP from the secondary interface in magenta) for association with each actin monomer. **c** Structural comparison of actin monomer-binding mechanisms of CARP domain of CAP1 (6fm2), profilin (2btf)^44^, gelsolin segment-1 (1eqy)^45^, ADF-H domain of twinfilin (3daw)^46^, and WH2 domain of ciboulot (1sqk)^47^. Front and side views of the complexes are shown. PDB entries are indicated in brackets

To reveal the principles of CAP–actin interactions, we crystallized the CARP domain of mouse non-muscle isoform, CAP1 (CAP1_317–474_) in complex with unmodified muscle ADP-G-actin. Crystals diffracted anisotropically to 2.3 Å in the c direction, and to 3.2–3.3 Å in the a and b directions (Supplementary Table 1, see Methods). The obtained crystal structure of a symmetric complex containing a dimer of the CARP domain bound to two ADP-actin monomers reveals several new and unexpected features (Fig. 1a, Supplementary Fig. 1a, and Supplementary Table 1). First, each actin monomer in the complex contacts each of the two subunits in the CARP homodimer, and each CARP monomer (within the homodimer) binds to two actin monomers using two distinct interfaces. This results in a sandwich-like structure, with the CARP homodimer squeezed between two ADP-actin molecules. The intertwined CARP domain dimer has an S-shape organization, in which the two ADP-actin molecules fit perfectly on both sides (Fig. 1a, b). Second, CAP interacts with actin unlike any other actin monomer-binding motifs that have been structurally characterized^44–47^. All of these other proteins bind to the “front side” or the barbed end interface of actin between subdomains 1 and 3, whereas the CARP domain binds to the “back side” on subdomains 1, 2, and 3 of actin (Fig. 1c). Third, the CARP domain binds G-actin through a much larger interface compared to other G-actin-binding proteins/domains. The large binding interface results from the two monomers within the CARP domain dimer interacting with actin monomers through different interfaces. The “primary interface” of the CARP domain on subdomains 1 and 3 of actin overlaps with the binding site of profilin on actin. However, the “secondary interface”, formed between the second CARP monomer and actin subdomain 2, is different from the interaction sites of other actin monomer-binding proteins characterized so far (Fig. 1a–c and Supplementary Fig. 1b). Analysis of the structures also revealed steric clashes between the CARP domain and profilin, as well as between the CARP domain and the twinfilin’s ADF-H domain (Supplementary Fig. 1g), providing a structural explanation for why these protein domains compete with each other for G-actin binding^20,34,43^.

**Fig. 2:**
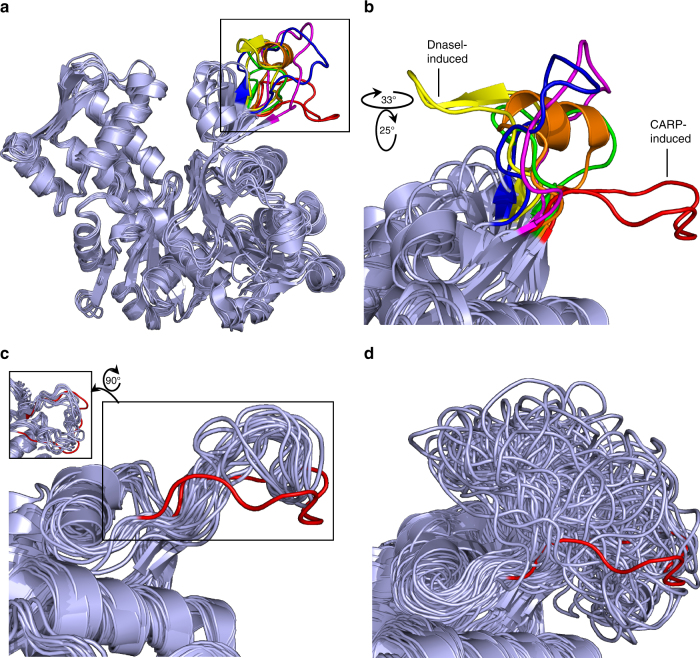
The D-loop of the G-actin adopts a unique conformation induced by the CARP domain. **a** Superimposition of selected structures of actin where the D-loop is resolved: un-complexed ADP-actin (1j6z)^48^ (orange); ADP-actin in complex with CARP domain (6fm2) (red); ATP-actin in complex with DnaseI (3w3d)^49^ (yellow); ATP-actin in complex with profilin (2btf)^44^ (magenta); ATP-actin with cytoD (3eks)^50^ (blue); ATP-actin in complex with ADF-H domain (3daw)^46^ (green). The conformation of the D-loop in CARP/ADP-actin monomer complex is different from other conformations reported. PDB entries are indicated in brackets. **b** A rotated, zoomed in view of the actin D-loop region from the structures listed in **a**. **c** Representative subset of D-loop conformations sampled with 10 ns intervals from the atomistic MD simulations of the ADP-actin–CARP domain complex (System 1, Supplementary Table 3) demonstrate the stability of the peculiar D-loop conformation detected in the crystal structure (in red). **d** A representative subset of D-loop conformations sampled with 10 ns intervals from the MD simulations of ADP-actin (isolated from ADP-actin–CARP domain complex, System 4, Supplementary Table 3) shows structural flexibility of the D-loop in the absence of the CARP domain

Although interactions with actin do not alter significantly the structure of the CARP domain (Supplementary Fig. 1c), the CARP domain induces a conformational change in the actin monomers. In the crystal structure, the D-loop of actin subdomain 2 has a pulled-back conformation and associates with the secondary interface of the CARP domain (Fig. 1a, b). This orientation is drastically divergent from all other D-loop conformations of actin reported so far^44,46,48–50^ (Fig. 2a, b, Supplementary Fig. 2a, and Supplementary Table 2). To confirm that the peculiar D-loop conformation does not result from crystal contacts (Supplementary Fig. 2b), we performed 1.2 μs all-atom MD simulations for the ADP-actin—CARP-domain complex (System 1, Supplementary Table 3) and for the ADP-actin isolated from this complex (System 4, Supplementary Table 3). The simulations demonstrate that the CARP homodimer stabilizes the D-loop of actin subdomain 2 in the extended conformation, while in uncomplexed ADP-actin this loop is dynamic and adopts a variety of conformations (Fig. 2c, d and Supplementary Fig. 2d). Compared to the structure of uncomplexed ADP-actin^48^, the CARP-bound ADP-actin monomer displayed also other small variations, which may be linked to the conformational change in the D-loop (Supplementary Fig. 1d–f, h). Together, the crystal structure and MD simulations reveal that the CARP homodimer binds to two ADP-actin monomers through a unique structural mechanism, and alters the conformation of the actin monomers.

### Interactions of CAP with ADP-G-actin and ATP-G-actin

**Fig. 3:**
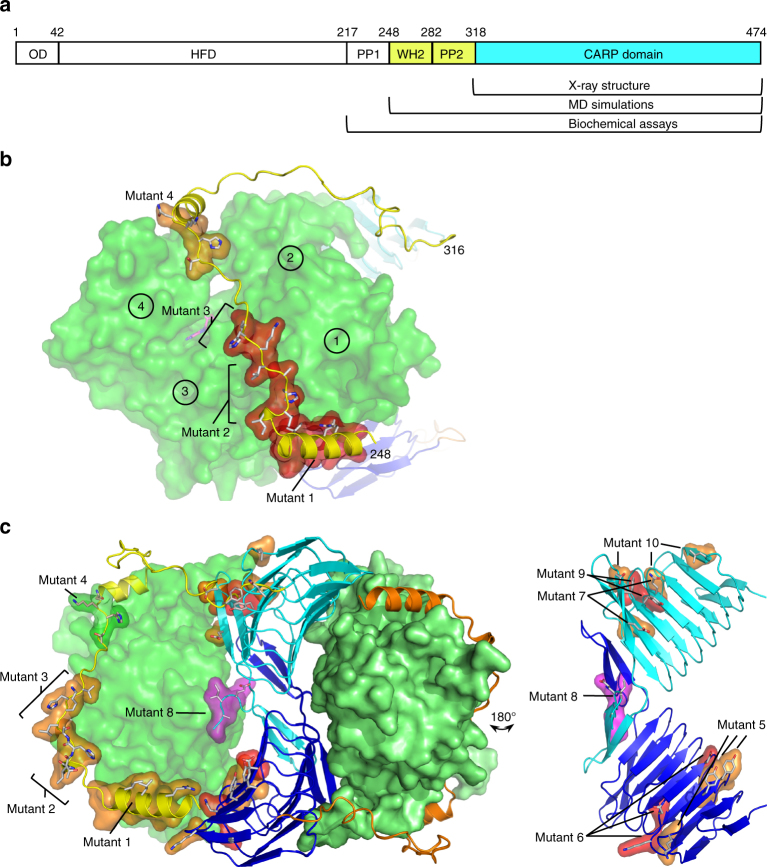
Structural mechanisms of ADP-G-actin and ATP-G-actin interactions with CAP1. **a** The domain architecture of CAP1. Oligomerization domain (OD), helical folded domain (HFD), polyproline region 1 (PP1), WH2 domain, polyproline region 2 (PP2), and CARP domain. The CAP1 regions in co-crystal structure and atomistic MD simulations model are indicated in cyan and in yellow, respectively. **b** MD simulation model of the ATP-actin–WH2 domain complex (System 3, Supplementary Table 3). The regions important for ATP-actin binding as determined by mutagenesis are highlighted in orange and red. Actin subdomains 1–4 are indicated by circles. **c** The ADP-G-actin–CAP1_248–474_ complex model (System 2, Supplementary Table 3) from MD simulations. The regions important for ADP-G-actin binding are indicated in orange and red, and the C-terminal tail of CAP1 is in magenta. For detailed effects of the mutants on actin binding, see Table 1 and Supplementary Fig. 4

We next performed mutagenesis and MD simulations experiments to test the roles of different CARP domain surfaces, and to reveal the mechanism by which the adjacent WH2 domain, which is unresolved in our structure (see Methods), contributes to actin monomer binding. We introduced four groups of mutations to the conserved clusters of residues in the WH2 domain of CAP1, and six groups of mutations in clusters of residues located at the primary and secondary actin-binding interfaces in our co-crystal structure (Table 1 and Fig. 3a–c). The mutant versions of the C-terminal fragment of mouse CAP1_217–474_ (C-CAP) were purified (Supplementary Fig. 3) and tested for ADP-G-actin and ATP-G-actin binding using a fluorometric NBD-actin assay^20,43^ (Supplementary Fig. 4). These experiments revealed that all four conserved clusters of residues in the WH2 domain are important for ATP-actin monomer binding, whereas none of the CARP domain mutations affected interactions with ATP-actin monomers. Consistent with the mutagenesis, MD simulations of the isolated ATP-actin–WH2 domain complex (System 3, Supplementary Table 3) revealed a stable association between the isolated WH2 domain (CAP1_248–295_) and an ATP-actin monomer (Supplementary Fig. 5a).

**Table 1:** Biochemical analysis of CAP1 mutants

			G-actin binding		Nucleotide exchange
	Mutant #		ATP-actin	ADP-actin	
		Wild type	++	++++	++++
WH2 domain	1	R253A L256A I260A	−	+++	++
	2	Δ^266^ITHA^269^	−	+++	+
	3	^270^LKHV^273^ → ^270^AAAA^273^	−	+++	−
	4	^279^THKN^282^ → ^279^AAAA^282^	+	++++	++++
CARP domain primary interface	5	K347A Y351A Y353A	++	+	+++++
	6	K365A N367A D372A	++	−	−
CARP domain secondary interface	7	D446A E449A	++	+	+++++
	8	Δ^471^EIAG^474^	++	+++++	++
	9	Y418A F447A	++	−	−
	10	L339A Q399A D446A	++	++	+++++

A summary table of C-CAP mutant affinities for ADP-G-actin and ATP-G-actin, and their effects on nucleotide exchange on ADP-actin monomers (see Supplementary Figs. 3c, d, 4). Symbols: *K*_d_ for ATP-actin: ++ (0.5–2 μM), + (2–5 μM), – (unmeasurable). *K*_d_ for ADP-actin: +++++ (0.005–0.025 μM), ++++ (0.025–0.1 μM), +++ (0.1–0.3 μM), ++ (0.3–1 μM), + (>1 μM), – (unmeasurable). Half-times of nucleotide exchange: +++++ (2–8s), ++++ (8–15s), +++ (15–19s), ++ (19–24s), + (24–28s), – (>29s)

While the CARP domain was dispensable for ATP-G-actin binding, mutations in both WH2 and CARP domains affected the ability of C-CAP to bind ADP-G-actin. Whereas mutations in the WH2 domain modestly decreased ADP-G-actin binding, some mutations in the CARP domain (e.g., K_365_A, N_367_A, and D_372_A in the primary interface) resulted in a complete loss of ADP-G-actin binding (Table 1, Fig. 3c, and Supplementary Fig. 3c). Importantly, mutations in both the primary (mutant 5 and mutant 6) and secondary interfaces (mutant 7 and mutant 10) were defective in ADP-G-actin binding, demonstrating that both interfaces of the CARP domain are required for interactions with actin. We then generated a MD simulation model of CAP1_248–474_ (System 2, Supplementary Table 3, and Supplementary Fig. 5b, c) combining homology-modeled WH2 domain with the crystal structure of the CARP–actin complex (see Methods). Consistent with the mutagenesis results, both the CARP domain and the N-terminal helix of the WH2 domain displayed stable association with ADP-G-actin in the 1.2 μs simulations (Supplementary Fig. 5d). Collectively, these results reveal the structural mechanisms by which CAP interacts with ATP-actin using its WH2 domain (Fig. 3b), and with ADP-G-actin using a combination of its WH2 and CARP domains (Fig. 3c).

### Mechanism of CAP-catalyzed nucleotide exchange

**Fig. 4:**
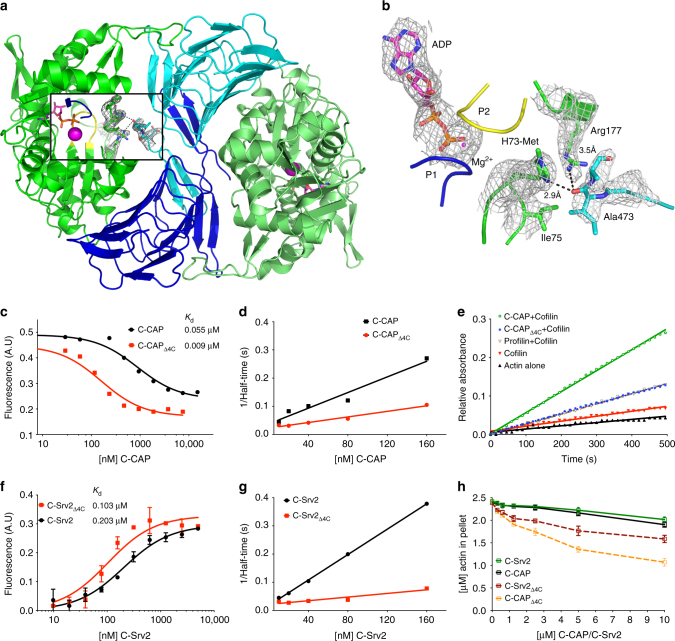
The C-terminal tail of CAP is critical for nucleotide exchange. **a** C-terminal tail of CAP1 in the crystal structure displayed in 2*F*_0_–*F*_C_ (*σ* = 1.0) electron density map. **b** Tail is positioned next to the nucleotide sensing region, loops P1 and P2 of actin that coordinate the nucleotide (*σ* = 1.0 in 2*F*_0_–*F*_C_ electron density map). **c** The affinity of C-CAP and C-CAPΔ4C for ADP-G-actin was determined by a fluorometric competition assay with NBD-labeled actin (0.18 μM) and the C-terminal ADF-H domain of mouse twinfilin (0.44 μM) (see Supplementary Fig. 4a). **d** A representative example of rate of ADP-G-actin (0.5 µM) nucleotide exchange in the presence of different concentrations of wild-type C-CAP and C-CAPΔ4C. **e** A representative example of actin filament turnover as followed by P_i_-release. F-actin (20 µM) was mixed with the indicated proteins (each 5 µM). **f** The affinity of C-Srv2 and C-Srv2Δ4C for ADP-G-actin (0.18 µM) was determined by fluorometric NBD assay. *n* = 3, error bars represent SD. **g** A representative example of rate of ADP-G-actin (0.5 µM) nucleotide exchange in the presence of C-Srv2 and C-Srv2Δ4C. **h** Monomer sequestering assay for C-CAP and C-Srv2 with different C-CAP/C-Srv2 concentrations using 2.5 µM actin. *n* = 3, error bars represent SD

One fascinating feature of the CARP domain—ADP-actin co-crystal structure is the close proximity of the C-terminal tail of the CARP domain with the “nucleotide state sensing region” of actin. The occupancy of third phosphate alters the hydrogen bonding network in the nucleotide-binding loops P1 and P2 of actin. These differences are relayed to methylated His_73_ located in the “sensing loop” that changes conformation between the two nucleotide states of actin^51^. Our structure revealed that the C-terminal tail of the CARP domain forms a hydrogen bond between a backbone oxygen of CARP Ala_473_ and the imidazole ring of actin His_73_ (Fig. 4a, b). Moreover, two other possible contacts between the C-terminal tail of CAP and the nucleotide sensing region of actin were revealed in MD simulations of the ADP-actin–CARP domain complex (System 1, Supplementary Table 3, Supplementary Fig. 6a), proposing that the C-terminal tail of CAP may contribute to nucleotide exchange on actin.

We generated mutant versions of mouse and budding yeast C-CAPs lacking the four C-terminal residues (Δ4C). Importantly, this same mutation in both proteins caused severe defects in nucleotide exchange on actin without compromising ADP-G-actin binding. Instead, the Δ4C mutants bound ADP-G-actin with higher affinity compared to the wild-type proteins (Fig. 4c, d, f, g, Supplementary Fig. 6b). Moreover, gel filtration analysis demonstrated that also in the context of full-length protein, Δ4C mutant does not disrupt ADP-G-actin binding activity of yeast Srv2 (Supplementary Fig. 6c). Importantly, deletion of the four C-terminal residues converted the C-terminal halves of mouse and yeast CAPs into actin monomer sequestering proteins (Fig. 4h), and severely compromised the ability of mouse C-CAP to enhance actin filament turnover in the presence of cofilin (Fig. 4e). These results suggest that deletion of the four C-terminal residues halts CAP’s normal progression, leaving it bound to ADP-actin but unable to convert monomers to the ATP-bound state, and therefore severely impairing actin filament turnover.

Another peculiar detail of the nucleotide exchange activity was revealed by the CARP domain mutants that bind to ADP-G-actin with weaker affinity. Whereas mutants in the CARP domain that completely disrupted ADP-G-actin binding lead to severe defects in nucleotide exchange, the CARP domain mutants displaying compromised, but still detectable, affinity for ADP-G-actin were slightly more efficient in promoting nucleotide exchange compared to the wild-type protein (Table 1 and Supplementary Fig. 3d). Thus, stable association between CAP and ADP-G-actin is not necessary for nucleotide exchange, at least under the in vitro conditions used in this assay.

### Physiological role of CAP-catalyzed nucleotide exchange

**Fig. 5:**
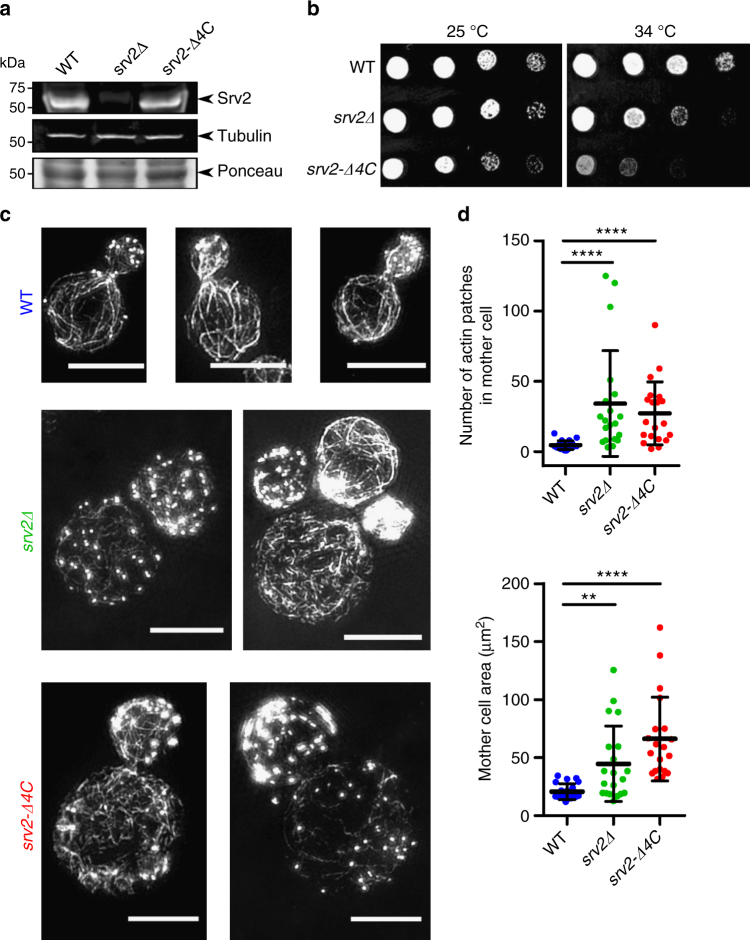
Truncation of Srv2/CAP’s C-terminal tail severely compromises actin cytoskeleton. **a** Western blots showing Srv2 expression levels in yeast strains with tubulin immunoblotting and Ponceau staining as loading controls. See Supplementary Fig. 7 for uncropped images. **b** Five-fold serial dilutions of yeast strains grown on YEPD plates at the indicated temperatures. **c** Structured illumination microscopy (SIM) of filamentous actin networks in yeast cells. Cells were grown in YEPD at 25 °C, fixed, and stained with Alexa488-phalloidin; scale bar, 5 µm. **d** Quantified differences in actin patch number and cell size across indicated yeast strains; *n* = 20 cells per strain; One-way, non-parametric ANOVA, *p* < 0.01 (**) and *p* < 0.0001 (****)

The ∆4C mutant described above enabled us to test the in vivo importance of CAP’s nucleotide exchange function, because the mutant does not disrupt actin binding. We integrated the *srv2-*Δ*4C* mutant at the *SRV2* locus of budding yeast, and verified that it is expressed at levels similar to wild-type Srv2 (Fig. 5a and Supplementary Fig. 7). Strikingly, the *srv2-*Δ*4C* mutant impaired cell growth, cell morphology, and actin organization as severely as a full deletion of the *SRV2* gene, despite missing only four residues located at its C-terminus (Fig. 5b–d). The *srv2*∆ and *srv2-*Δ*4C* mutants both caused a dramatic increase in the size of mother cells, a depolarization of cortical actin patches, and loss of normal actin cable staining. These results suggest that CAP’s nucleotide exchange activity plays a critical role in recharging actin monomers in vivo.

## Discussion

**Fig. 6:**
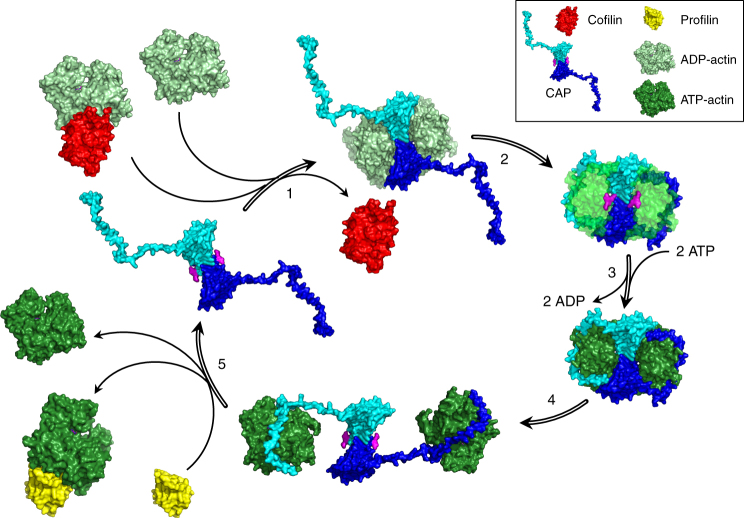
A working model for how CAP catalyzes nucleotide exchange on actin monomers in cells. (1) CAP can interact with both free and cofilin-bound ADP-G-actin using CARP domain. This interaction puts the WH2 domain in position to competitively replace cofilin, leading to cofilin dissociation from the ADP-actin monomer, as previously observed biochemically^20^. (2) The tight association of both CARP and WH2 domains with ADP-actin monomers, together with the penetration of the C-terminal tail of CAP into the nucleotide-binding pocket, catalyzes a change in conformation and dynamics of the ADP-actin monomer to enhance the rate of nucleotide exchange. (3) Nucleotide in actin is rapidly exchanged from ADP to ATP. (4) The CARP domain has little if any affinity for ATP-actin, leaving dimeric CAP molecules associated with ATP-G-actin solely through their WH2 domains. (5) Profilin has high affinity for ATP-actin monomers, and binds directly to the PP1 domain of CAP^31^, adjacent to the WH2 domain. Thus, as ATP-actin monomers dissociate from CAP, they are rapidly bound by profilin, replenishing the pool of ATP-actin monomers available for assembly. This leaves CAP primed for the next round of nucleotide exchange

A model for CAP-catalyzed re-charging of actin monomers is presented in Fig. 6. In cells, ADP-actin monomers either dissociate spontaneously from the filament pointed ends or their depolymerization is enhanced by the ADF-H domain proteins ADF/cofilin and twinfilin^12,13,28^. The C-terminal half of CAP efficiently catalyzes nucleotide exchange on both uncomplexed and ADF/cofilin-bound ADP-actin monomers^22,23,34^. We demonstrate that the dimeric CARP domain associates with these newly depolymerized ADP-actin monomers through a unique structural mechanism that involves two separate CARP domain surface regions, which collectively interact with the “back side” of actin on subdomains 1, 2, and 3. The CARP domain specifically binds ADP-actin monomers, whereas the isolated WH2 domain of CAP displays no appreciable binding preference for ADP-actin vs. ATP-actin monomers^20,34,43^. Because ADP-actin and ATP-actin monomers do not display drastic structural differences^48^, future work is required to reveal why CARP domain specifically associates with ADP-G-actin while the WH2 domain of CAP binds both ADP-actin and ATP-actin monomers. Nevertheless, our data suggest that CAP combines its WH2 and CARP domains to achieve high-affinity interactions with ADP-actin monomers, and to promote the dissociation of ADF/cofilin from ADP-actin monomers. Following the initial interaction of the CARP domain dimer and ADP-actin monomers, the WH2 domains “embrace” the two ADP-actin monomers leading to a formation of a compact complex, where nucleotide exchange of actin occurs. The “re-charged” ATP-actin monomers remain transiently bound to the WH2 domains of CAP, and then are released spontaneously, or transferred to profilin, which binds to the adjacent PP1 poly-proline region in CAP^31^.

Our experiments provide evidence that three structural features of the CAP/ADP-G-actin complex are important for nucleotide exchange. First, we showed that the C-terminal tail of the CARP domain, which “penetrates” into the actin molecule and associates with its “nucleotide sensing region”, is important for efficient “re-charging” of actin by CAP. Second, we found that the CARP domain induces a unique conformational change in the D-loop of actin. Importantly, subtilisin-cleavage of the D-loop of ADP-actin monomers results in ~3-fold increase in the rate of ADP-to-εATP nucleotide exchange on actin (Supplementary Fig. 2c), similar to what was previously reported for ATP-to-εATP exchange on subtilisin-cleaved actin^52^, suggesting that the D-loop communicates allosterically with the nucleotide-binding pocket. However, in contrast to the mechanism by which Sos guanine-nucleotide-exchange-factor promotes GDP-for-GTP exchange on Ras GTPase, we did not detect major conformational changes in the nucleotide-binding pocket of actin^53^. Third, the presence of functional WH2 domain is important for efficient nucleotide exchange by mouse CAP1. Consistent with previous structural work^47,54,55^, our MD simulations suggest the WH2 domain of CAP binds to the cleft between actin subdomains 1 and 3. The precise mechanism by which the WH2 domain contributes to nucleotide exchange remains to be elucidated. However, MD simulations provided evidence that association with the CARP domain increases the dynamics of ADP-G-actin leading to an opening of the cleft between subdomains 2 and 4, and that these features are further augmented in the presence of the WH2 domain (Supplementary Fig. 8). It is also important to note that the role of the WH2 domain in nucleotide exchange varies between different species. Although malaria parasite CAP, which is entirely composed of a CARP domain, efficiently catalyzes nucleotide exchange on actin^43^, mouse and budding yeast CAPs cannot efficiently promote nucleotide exchange without functional WH2 domain, especially in the presence of cofilin^27,56^.

Our in vivo work using the yeast *srv2-*Δ*4C* mutant provides the first direct evidence that CAP-catalyzed nucleotide exchange is critical for actin cytoskeleton organization and function. Earlier genetic evidence from yeasts suggested that nucleotide exchange catalyzed by profilin may be important in vivo^57,58^. However, the mutants used in these studies all weakened actin-binding affinity, and thus the in vivo role of profilin in nucleotide exchange remains to be tested in the manner we have demonstrated here for CAP, with a mutation that disrupts nucleotide exchange without weakening actin affinity. Two other observations calling into question whether profilins catalyze nucleotide exchange in vivo are that profilins do not bind ADP-G-actin with high affinity^19^, and profilins are much less efficient than CAP in accelerating actin filament turnover in the presence of cofilin (Fig. 4e and ref. ^22^). Whether profilin has partially redundant roles with CAP in promoting nucleotide exchange in cells, or instead is required primarily to maintain homeostasis in distributing actin monomers between formin-dependent and Arp2/3 complex-dependent actin assembly pathways, remains to be determined^59,60^.

Collectively, our study uncovers the molecular mechanism by which the WH2 and CARP domains of CAP associate with actin monomers and accelerate nucleotide exchange on ADP-actin. However, it is important to note that in most eukaryotic organisms, including yeast and mammals, CAP oligomerizes into hexameric complexes that have additional functions in accelerating ADF/cofilin-dependent and twinfilin-dependent actin filament disassembly, which depend on the N-terminal helical folded domain^21,26–28^. Thus, in the future it will be important to reveal how the different activities of CAP are structurally and functionally coordinated between its N-terminal and C-terminal functional units.

## Methods

### Proteins

CAP1_242–474_ for crystallization experiments was expressed in BL21(DE3) *E. coli* (Sigma-Aldrich) as a 10xHis-3C fusion protein using pCoofy18 vector, a kind gift from Sabine Suppmann (Addgene plasmid #43975). After 20 h of cultivation at +22 °C in LB auto-induction media (AIMLB0210, Formedium), cells were collected by centrifugation and suspended to lysis buffer (50 mM Tris-HCl, 150 mM NaCl, 25 mM imidazole, pH 7.5) containing protease inhibitors (200 µg/ml PMSF, 1 µg/ml leupeptin, 1 µg/ml aprotinin, 1 µg/ml pepstatin A, 20 µg/ml Dnase I; all from Sigma-Aldrich). Cells were homogenized by EmulsiFlex—C3 (Avestin Inc.) and the supernatants were clarified by centrifugation. Supernatant was loaded to equilibrated HisTrap FF crude 5 ml prepacked column (GE Healthcare) coupled to ÄKTA Pure chromatography system, washed 20xCV (column volume) with 1:5 ratio of elution buffer (50 mM Tris-HCl, 150 mM NaCl, 250 mM imidazole, pH 7.5) and lysis buffer, and eluted using an imidazole gradient. Protein-containing fractions were pooled and dialyzed (50 mM Tris-HCl, 150 mM NaCl, 1 mM DTT, pH 8.0) O/N at 4 °C containing ~1:100 3C protease in SnakeSkin dialysis tubing (Thermo Scientific). Equilibrated Ni-NTA beads (Qiagen) were added to the sample and incubated 1 h at 4 °C rotating to remove any non-cleaved protein. Supernatant was collected and concentrated by centrifugation with Amicon Ultra-4 10 kDa centrifugal filter (Merck). Finally, the sample was further purified by gel filtration using a HiLoad 16/600 SD 200 pg (GE Healthcare) column pre-equilibrated with 5 mM HEPES, 50 mM NaCl, 0.2 mM DTT, 0.01% NaN_3_, pH 8.0. The peak fractions were collected and concentrated to 5.4 mg/ml and stored on ice for further use or were flash frozen in liquid N_2_ for long-term storage.

For biochemical experiments, mutations to C-CAP were generated using primers shown in Supplementary Table 4. Expression of the wild-type and mutant proteins (both mouse and yeast) was performed as above in one liter scale. Cells were suspended to a lysis buffer containing a Complete Ultra protease inhibitor cocktail (Roche) and stored at −80 °C by snap freezing. For protein purification, cell suspensions were thawed in a water bath and disrupted by sonication. Cell lysates were clarified by centrifugation and batch purified by adding 1 ml of Ni-NTA agarose beads (Qiagen) for 2 h at 4 °C rotating. Beads were washed with wash buffer (50 mM Tris-HCl, 150 mM NaCl, 50 mM imidazole, pH 7.5) for 40xCV and suspended to cleavage buffer (50 mM Tris-HCl, 150 mM NaCl, 25 mM imidazole, 1 mM DTT, pH 8.0) for O/N cleavage with ~0.01 mg/ml of 3C protease at 4 °C. Supernatant was collected and concentrated to 0.5 ml volume with Amicon Ultra-4 10 kDa centrifugal filter (Merck) and the proteins were further purified by gel filtration (SD200 Increase 10/300, GE Healthcare) in 2 mM Tris-HCl, 100 mM KCl, 1 mM MgCl2, 0.1 mM CaCl2, 0.1 mM DTT, pH 8.0. Protein-containing fractions were concentrated as above stored by snap-freezing with liquid N_2_ at −80 °C. Mutants used for biochemical assays eluted in similar volumes in the gel filtration, those which eluted in void fraction were discarded from further analysis.

Cofilin and profilin were purified as previously described^20,43^. Muscle α-actin was prepared from rabbit muscle acetone powder (Pel Freez) as previously described^20^ and stored at 2 mg/ml by snap-freezing with liquid N_2_ at −80 °C.

Full-length Srv2 proteins were expressed as GST-tagged fusion proteins in pGAT2 vector. Plasmids were transformed into BL21(DE3) *E. coli* and protein expression was performed at 37 °C in 2xLB media by IPTG induction at OD_600_ of 0.5–0.7. Expression was continued at 16 °C for 24 h. Cells were disrupted by sonication and supernatants clarified by centrifugation. Equilibrated glutathione agarose beads (Thermo Scientific) were added to the sample and incubated 2 h at 4 °C. Beads were washed with wash buffer (50 mM Tris-HCl, 150 mM NaCl, pH 8.0) for 20xCV and protein was eluted with 4xCV in a gravity column with elution buffer (100 mM Tris-HCl, 100 mM NaCl, 20 mM reduced glutathione, pH 8.8). Elution fractions were pooled and loaded to HiTrap Q HP (GE Healthcare) anion exchange column equilibrated in Buffer A (100 mM Tris, 100 mM NaCl, pH 8.8). Column was washed with 5xCV of Buffer A and then 5xCV with 20% Buffer B (100 mM Tris, 400 mM NaCl, pH 8.8). Finally, protein was eluted with a salt gradient, peak fractions were pooled and concentrated with Amicon Ultra-4 50 kDa centrifugal filter (Merck) and stored as described above.

### Crystal structure of CAP1_317–474_–ADP–actin complex

For complex formation with mouse CAP1_242–474_, actin was thawed and prepared by first exchanging Ca^2+^ metal to Mg^2+^ during O/N dialysis (5 mM HEPES, 0.2 mM MgCl2, 0.2 mM EGTA, 0.2 mM ADP, 0.2 mM DTT, pH 8.0) at 4 °C. The actin was then treated with 20 U/ml hexokinase (Sigma-Aldrich) and 0.3 mM glucose for 1 h and mixed with CAP1_242–474_ in ~1:1 molar ratio. Complex was further purified by gel filtration (SD200 Increase 10/300, GE Healthcare), pre-equilibrated with 5 mM HEPES, 50 mM NaCl, 0.2 mM ADP, 0.2 mM MgCl2, 0.2 mM DTT, pH 8.0. Major peak was collected and concentrated to 6 mg/ml as above. The sample was immediately set up for crystallization with sitting drop method (1:1 ratio in 200 nL drop, Mosquito, TTP) at 20 °C, at the Crystallization Facility (Institute of Biotechnology, Helsinki). After 24 h, a single crystal appeared in Helsinki Complex screen in a well with 0.1 M Tris-HCl, 0.2 M LiCl, 20% (w/v) PEG8000, pH 8.0 of mother liquid. Crystallization conditions were further optimized; however, only a few crystals could be obtained after numerous attempts at 0.1 M Tris-HCl, 0.2 M LiCl, 20–23% (w/v) PEG8000, pH 7.9–8.5. For data collection, crystals were snap frozen in liquid N_2_ by fishing directly from 96-well plates and soaking for cryo-protection in 25% glycerol (v/v) containing mother liquid. Diffraction data were collected at Diamond Light Source synchrotron (Didcot, UK) at 100 K on I04 beamline with Pilatus3 6M detector. A complete data set was obtained at 0.97 Å wavelength with 0.1° oscillation angle per diffraction image with a total of 1400 images collected. Data were integrated using X-ray Detector Software^61^, merged and scaled with AIMLESS (CCP4^62^). The initial molecular replacement solution of 1:1 complex CARP-domain and actin was obtained with BALBES^63^ program, part of CCP4 suit, giving best result (*Q* factor = 0.6809) using 1k4z model for CARP domain and 3tpq for the actin with Rwork/Rfree = 0.3470/0.4470, after single REFMAC^64^ refinement round. Rounds of manual model building in COOT^65^, introduction of translation-libration-screw grouping and refinement with BUSTER^66^ lead to final model with *R*_work_ = 0.186 and *R*_free_ = 0.234. It is important to note that we used a CAP1 construct (CAP1_242–474_) composed of the WH2 domain and the CARP domain in the crystallization trials, but electron density was observed only for the CARP domain (residues 317–473) and actin, whereas the first 74 residues of the CAP1_242–474_ were absent from the structure. Mass spectrometry analysis of the crystallization drops confirmed that the flexible N-terminal region of CAP1_242–474_ construct, containing the WH2 domain, had a tendency to degrade thus resulting in a product of a size of the CARP domain that was seen in the final electron density maps. The diffraction data was strongly anisotropic (CC_1/2_ > 0.3 at 2.3 Å along *l-*axis, 3.2 Å along *hk* plane) with Δ*B* of 56.16 Å^2^ between the axes which might explain a high overall *B*-factor (Supplementary Table 1) when using individual B-factor refinement in the final model. Despite high average *B*, distribution of *B*-factors along the structure was normal. We considered anisotropic treatment of the merged data using ellipsoidal cut (STARANISO) which in our model lowers the average *B*-factor from ~129 to ~75 Å^2^ (Wilson *B* from 86 to 55 Å^2^), normal for the resolution range. However, ellipsoidal cut decreased slightly the completeness of our data in the 3.0–3.6 Å resolution range. This caused the electron density maps to be slightly discontinuous in some parts of CARP, but allowed us to observe more high-resolution details overall in the electron density maps. For these reasons, anisotropically treated data was only used to polish and finalize the final model, especially in the placement of waters and some side chains. Finally, we refined the model against either of these data sets yielding nearly identical models (RMSD ~0.5). Before deposition to PDB, the model was refined to *R*_work_/*R*_free_ 18.6%/23.4% using non-treated data at 2.8 Å with *I/σI* = 1.3 as resolution cutoff criteria.

### Biochemical experiments

To determine binding of C-CAP proteins to actin with 4-chloro-7-nitrobenzofurazan (NBD) assay, NBD-actin was prepared as previously described^43^ from rabbit skeletal muscle. NBD-ADP-actin was prepared by treating 30 μM NBD-actin with hexokinase (20 units/ml) in exchange buffer (2 mM Tris-HCl, 0.1 mM MgCl_2_, 0.2 mM EGTA, 0.2 mM ADP, 0.3 mM glucose, 0.1 mM DTT, pH 8.0) at +4 °C for 3 h. For assays with NBD-ATP-actin, the protein was similarly converted from Ca^2+^ to Mg^2+^-form by incubating 5 min in exchange buffer without hexokinase. For determination of protein concentration at A_290_ (*ε* = 26,600 M^−1^ cm^−1^), both ADP-actin and ATP-actin were buffer exchanged to 2 mM Tris-HCl, 0.1 mM MgCl_2_, 0.2 mM EGTA, 0.2 mM ADP/ATP, 0.1 mM DTT, pH 8.0 using Zeba Spin Desalting columns (Thermo Scientific) and centrifuged at 100,000 rpm with TLA-100 rotor (Beckman) for 5 min at 4 °C. The affinities of mouse C-CAP constructs for ADP-actin and ATP-actin were determined as previously described^43^. Affinity of C-Srv2 proteins for ADP-actin was determined as previously described^20^. Samples were measured on a 96-well microtiter plate for fluorescence at 482/520 nm using Varioskan Lux plate reader (Thermo Scientific) with 1000 ms exposure at +25 °C. The fluorescence data were fitted assuming one site competition for ADP-actin assuming 0.03 μM affinity for twinfilin^43^ and one site saturating binding for ATP-actin and C-Srv2 proteins. Equations used for the fitting of the data are described in Graphpad 7 manual (https://www.graphpad.com/guides/prism/7/curve-fitting/index.htm?reg_binding-saturation.htm).

For nucleotide exchange assays, Ca^2+^-ATP-actin was first exchanged to Mg^2+^-ATP-actin by using exchange buffer described above. ADP-actin was prepared in dialysis at +4 °C for 3 h (5 mM Tris-HCl, 0.1 mM MgCl2, 0.1 mM EGTA, 0.2 mM ADP, 0.3 mM glucose, pH 8.0) with 20 U/ml hexokinase and 0.3 mM glucose present to remove ATP traces from the solution. ADP-actin was then centrifuged at ~386,000 × *g* for 20 min, and concentration determined by A_290_ against dialysis buffer. C-CAP proteins and ~30 μM ADP-actin were diluted to buffer A (20 mM HEPES, 0.1 mM MgCl_2_, 0.1 mM DTT, pH 7.4) and reaction was started by adding buffer B (20 mM HEPES, 1.9 mM MgCl2, 160 mM KCl, 0.1 mM DTT, 50 μM ε-ATP (Jena Biosciences), pH 7.4) in 1:1 ratio, mixed and followed at fluorescence spectrophotometer (Agilent) at 360 nm/412 nm (excitation/emission) until saturation at +22 °C. Data were fitted assuming one phase association and half-times were calculated from the fit. For the titration experiments carried out with different Srv2/CAP concentrations, the assay was done twice for each mouse CAP concentration and one time for each yeast Srv2 concentration.

For cleavage of ADP-actin with substilisin, actin was prepared as described above in dialysis. Substilisin (Sigma-Aldrich, P5380) was added in 1:100 molar ratio to ADP-actin. Cleavage was continued for 2 h on ice and measured for nucleotide exchange as above. Completeness of the cleavage confirmed on SDS-PAGE was >90%.

Actin filament turnover assay was performed by following the release of inorganic phosphate with 2-amino-6-mercapto-7-methylpurine ribonucleoside (MESG) and purine-nucleoside phosphorylase (PNP) (EnzChek, Thermo Scientific). Rabbit muscle actin was mixed with 0.2 mM MESG and 6 units of PNP in G-buffer and polymerization was initiated with 20 mM KCl/NaCl and 2 mM MgCl_2_. After 10-min incubation, reactions with 5 μM protein(s) and 20 μM actin were prepared and transferred on a 96-well microtiter plate. The release of phosphate was followed for 30 min at 360 nm using Varioscan Lux multimode microplate reader (Thermo Scientific) at 25 °C. The assay was repeated two times for each combination of proteins.

For actin cosedimentation assay, actin was polymerized in 20 mM Tris pH 7.5, 100 mM KCl, 0.2 mM EGTA, 1 mM MgCl2, 1 mM ATP, 0.2 mM DTT for 30 min at 22 °C. Different amounts of C-CAP proteins were added to the mixture of 2.5 μM actin and incubated 30 min at 22 °C. Samples were centrifuged at 100,000 rpm for 30 min with TLA-100 rotor (Beckman) to pellet the F-actin. Samples from supernatant and pellet were analyzed on SDS-PAGE, imaged with ChemiDoc XRS+ imaging system (BioRad) and quantified using Image Lab (BioRad).

The thermal stability of the proteins was measured by Thermofluor assay (known also as Thermal Shift Assay) in the nucleotide exchange buffer. Proteins were diluted to 25 µM, and the SYPRO Orange dye (Thermo Scientific, #S6650) was diluted 300 times with the nucleotide exchange buffer prior to reaction. Reactions of 25 µl were prepared in 96-well PCR plate, consisting of 2.5 µl diluted protein, 2.5 µl of diluted SYPRO orange dye, and 20 µl of the nucleotide exchange buffer. The change of fluorescence as an indication for protein denaturation was measured on Mx30005P qPCR instrument (Agilent) every 30 s with a rate of 1 °C/ 30 s. The midpoint values for unfolding were taken as a value for thermal stability.

Binding of full-length Srv2 proteins to rabbit muscle ADP-actin was analyzed by size-exclusion chromatography. Preparation of ADP-actin was performed as described for nucleotide exchange assays. SD200 Increase 10/300 (GE Healthcare) was equilibrated in 5 mM HEPES, 100 mM NaCl, 0.05 mM ADP, 0.05 mM MgCl2, 0.5 mM β-mercaptoethanol, and retention of ADP-actin was first analyzed by loading 300 μl of 10 μM ADP-actin to the column. Then, retention was analyzed for 10 μM ADP-actin mixed with 10 μM GST-Srv2, or with 10 μM GST-Srv2-Δ4C. Peak fractions were collected and analyzed by SDS-PAGE.

### Atomistic MD simulations

The WH2 domain of the CAP1 was obtained by homology modeling based on three structures (PDB entries 1sqk^54^, 2a3z^47^, and 4pl7^67^) using the comparative modeling protocol of Rosetta (RosettaCM)^68^. This initial model of WH2 was used for MD simulations of ATP-actin-WH2 (System 3, Supplementary Table 3). The model was further extended by adding PP2 and connecting it to the CARP domain from our structure. There were two possible ways of connecting WH2 to CARP domain: in *cis* (Supplementary Fig. 5b) or in *trans* (Supplementary Fig. 5c). The *cis* configuration requires PP2 to stretch across a distance that is at least 11 Å longer than in the *trans* configuration, making it both sterically and entropically unfavorable. The *trans* configuration has no steric clashes and in addition allows the regulatory region PP2 to be accessible, and thus represents more plausible model (Supplementary Fig. 5c). One thousand decoys of the ADP–actin–CAP1_248–474_ dimeric complex were generated. The models were ranked based on their total score, and the top ranking five models were selected to initiate atomistic MD simulations.

The structures of protein molecules (actin, CARP domain, WH2, and CAP1_248–474_) were prepared using Propka^69^ (p*K*_a_ estimation based on the crystallized complex and determination of protonation states at pH = 6.8), Chimera^70^ (placing hydrogens), VMD^71^ (protein structure building and visualization), and PyTopol (protein topology conversion from CHARMM to the GROMACS format). In all systems, E454 and H416 of the CARP domain were protonated; N-terminus of actin was acetylated; the His73 residue of actin was methylated (parameters were obtained by analogy); and the crystal water molecules and the bound Mg ion were kept. In each system containing ATP-actin, ATP was docked into the binding pocket based on the ADP position. Each system containing the WH2 domain was initiated from the top scoring five Rosetta models as described above.

All simulations were carried out using GROMACS 5.1^72^ employing the Charmm36 force field^73^ for the proteins and the TIP3P model^74^ for water. The equations of motion were integrated using a leap-frog algorithm with a 2 fs time step. All bonds involving hydrogens were constrained using the LINCS algorithm^75^. Long-range electrostatic interactions were treated by the smooth particle mesh Ewald scheme^76^ with a real-space cutoff of 1.2 nm, a Fourier spacing of 0.12 nm, and a fourth-order interpolation. A Lennard–Jones potential with a force-switch between 1.0 and 1.2 nm was used for the van der Waals interactions.

Each protein complex was placed in a rhombic dodecahedral simulation box maintaining at least a distance of 15 Å to the box sides and solvated in 0.15 M NaCl solution, ensuring neutrality of the system. Before production runs, steepest descent minimization and successive equilibration simulations (in total ~500 ps) in the NVT and NPT ensembles using the Berendsen thermostat and barostat^77^ were performed. In these simulations, initially all protein heavy atoms, and later only the C_α_ atoms were restrained with a force constant of 1000 kJ/mol/Å^2^; the time step was then ramped up from 1 to 2 fs. The number of water molecules and the average box volume at the start of the production runs are given in Supplementary Table 3.

For production runs, each system was simulated in the NPT ensemble for ~1.2 µs. Five independent repeats were performed for each system (Supplementary Table 3). The total time scale covered in the simulations was >24 μs. Protein-ADP/ATP-Mg complex, and solvent (water and NaCl) were coupled to separate temperature baths at 310 °K using the Nosé-Hoover thermostat^78,79^ with a time constant of 1.0 ps. Isotropic pressure coupling was performed using the Parrinello–Rahman barostat^80^ with a reference pressure of 1 atm, a time constant of 5 ps, and a compressibility of 4.5 × 10^−5^ bar^−1^. All analyses were performed separately for each simulation repeat. The averages and standard deviations over independent repeats are reported.

### In vivo experiments with yeast

All strains are in the s288c background from BGY311 (*MATα*, *his3*Δ*200*, *leu2-3*,*112*, *ura3-52*, *trp1-1*(*am*), *lys2-801*(*oc*)). Mutant yeast strain *srv2*Δ*::HIS3* (BGY330) was generated previously^30^. To generate a mutant strain with the C-terminal four amino acid deletion, we introduced an early stop codon using site-directed mutagenesis in a previously generated Srv2 integration plasmid, pBG861^30^. The resulting plasmid, pBG1951, was sequenced and then used to generate *srv2-*Δ*4C::TRP1* (SGY045) by homologous recombination. The presence of the *srv2-*Δ*4C* mutation, replacing wild-type *SRV2* in the genome, was confirmed by PCR analysis of isolated genomic DNA.

To measure Srv2/CAP protein levels in yeast cells, strains were grown in 10 ml cultures of YEPD at 25 °C to log phase, collected by centrifugation at 3000 × *g* for 2 min, and resuspended in 20% TCA (tricholoracetic acid) at 25 °C. Cells were centrifuged at 16,000 × *g* for 30 s, and the pellet was resuspended with 20% TCA, and vortexed with glass beads for 7 min. The mixture was diluted to final 5% TCA, and centrifuged at 3000 × *g* for 10 min. Pellets were neutralized with 1 M Tris 8.0, resuspended in Laemli buffer (150 mM Tris-HCl (pH 6.8), 300 mM DTT, 6% SDS, 0.3% bromophenol blue, 30% glycerol, and 12% beta-mercaptoethanol), and immunoblotted. Blots were incubated for 1 h with either 1:500 primary chicken anti-α-Srv2 (Batch number 3491, Aves Labs, Inc., Tigard, OR) or 1:1000 primary mouse anti-α-tubulin antibody (sc-32292; Santa Cruz Biotechnology), then washed and probed with secondary anti-chicken antibody (IRDye800CW, #603-131-126, LI-COR Biosciences) for Srv2 or anti-mouse antibody (IRDye-680, #926-32220,0 LI-COR Biosciences) for Tubulin. Blots were imaged on an Odyssey gel scanner (LI-COR Bioscience).

To visualize the actin cytoskeleton in cells, yeast strains were grown in YEPD to log phase and fixed with formaldehyde (4% final) for 30 min at 25 °C. Fixed cells were washed with 1× PBS and stained with AlexaFluor^TM^-488-Phalloidin (Thermo Scientific) overnight at 4 °C. Cells were then washed three times with 1× PBS, and mounted on slides immediately before imaging. Cells were imaged on a Nikon N-SIM (Structured Illumination Microscopy) instrument (Nikon Instruments, Melville, NY) equipped with a SR Apo TIRF AC 100xHx1.49 N.A. oil immersion objective, a LU-N3-SIM laser unit, and an ORCA-flash4.0 CMOS camera (Hamamatsu Photonics, Boston, MA). 3D-SIM image stacks were acquired with a *Z*-interval of 0.1 µm for a 0.9 µm section at the central plain of the cell. Images were captured with 500 ms exposure time. Fifteen raw images were acquired per *Z*-position, and reconstruction of images were performed using the reconstruction slice method from NIS-Elements software (Nikon Instruments). Final images were then analyzed in ImageJ (NIH) to quantify actin patch number in mother cells and mother cell size, and the data were plotted in Graphpad Prism 6.

### Quantification and statistical analysis

The statistical details, number of experiments and statistical analyses, are described in the figure legends or the Methods in all relevant cases. Softwares for quantification and data analysis are indicated in experimental details in the Methods section.

### Data availability

The WH2-PP2-CARP model(s) obtained from atomistic simulations, together with the raw data, are available in Zenodo. The crystal structure has been deposited to the Protein Data Bank (PDB) under access code 6fm2. All relevant experimental data are available upon a reasonable request from the corresponding author.

## Electronic supplementary material

Supplementary Information

Peer Review Report
