# Supplementary material for: Structural basis of actin monomer re-charging by cyclase-associated protein
Source: Nat Commun. 2018 May 14;9:1892. doi: 10.1038/s41467-018-04231-7 (PMC5951797; doi:10.1038/s41467-018-04231-7)
Supplement: Supplementary file 1 — Supplementary Information [file 41467_2018_4231_MOESM1_ESM.pdf]

Structural basis of actin monomer re-charging by cyclase-associated protein

Kotila *et al.*

Supplementary Figure 1

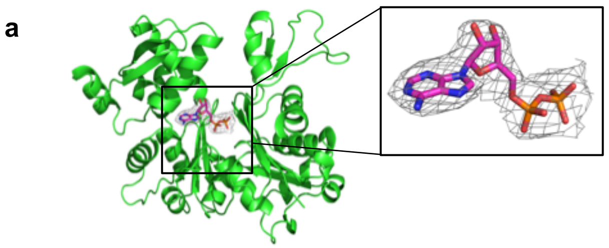

b

PDB	Protein	Interface (Å²)	Tot. actin surface (Å²)
2btf	Profilin	1039	16778
1eqy	Gelsolin	1078	16132
3daw	Twinfilin	1157	17136
1sqk	WH2	854	16271
6fm2	CARP	**1939**	17099

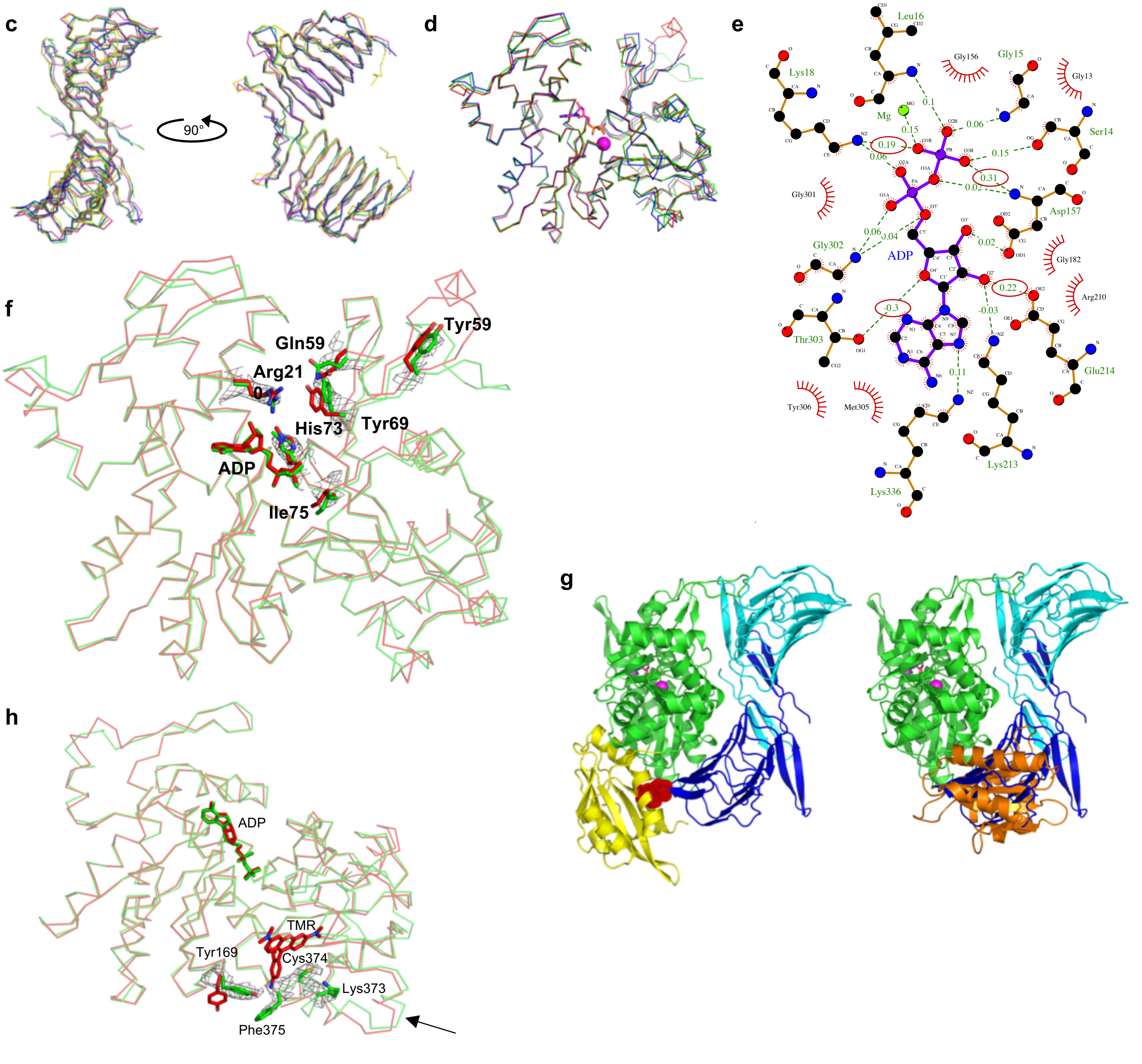

Supplementary Fig. 1. Crystal structure of the CARP domain-ADP-actin complex. (a) Electron density of ADP in actin in our crystal structure (2F0 - FC, contoured at $\sigma = 1.0$). **(b)** Comparison of binding surface areas of different actin-binding proteins calculated with PISA[80]. (c) Comparison of crystallized CARP domains displayed as a ribbon model (for clarity) using Pymol: 2b0r (yellow) Cryptosporidium parvum, 1k4z (magenta) Saccharomyces cerevisiae, 1k8f (green) Homo sapiens, and Mus musculus CARP domain model from our actin complex (blue). **(d)** Comparison of ADP-actin crystal structures superimposed on subdomain 3 (residues 145-180 and 270-337). CARP-actin (green), un-complexed actin rabbit muscle actin (1j6z, red), and non-polymerizable Drosophila melanogaster actin (2hf3, blue). A major conformational change is observed mainly in the D-loop located in subdomain 2. **(e)** Analysis of ADP ligand interaction and bond distances in the nucleotide binding site with Ligplot+ v1.4.581. Distances are shown as a difference to un-complexed ADP-actin (1j6z). Negative values indicate more distant than in 1j6z; most apparent differences are indicated as red circles. Please note that our structure has Mg^{2+} ion, and 1j6z has Ca^{2+} ion. **(f)** Comparison of side chain movements in actin subdomains 2 and 4 displayed in electron density (2F0 - FC, contoured at $\sigma = 1.0$). Structures are aligned on the subdomain 3. Shown here are un-complexed actin (1j6z, red) and actin from the CARP domain complex (green). **(g)** On the left, steric clash (red) between actin-bound twinfilin's ADF-H domain (yellow) (3daw) and the CARP domain (blue). On the right, profilin (orange) (2btf) overlaid with the CARP domain (blue) on actin (green). (h) C-terminal residues of actin (Lys373, Cys374 and Phe375) are visible in our CARP domain/actin complex (2F0 - FC, contoured at $\sigma = 1.0$). A shift in Tyr169 is observed when compared to un-complexed actin (red), most likely due to absence of TMR in our structure. Thus, formation of π-π stack with Phe375 is plausible. In addition, a clear movement in α-helix359-365 is visible (pointed with an arrow).

Supplementary Figure 2

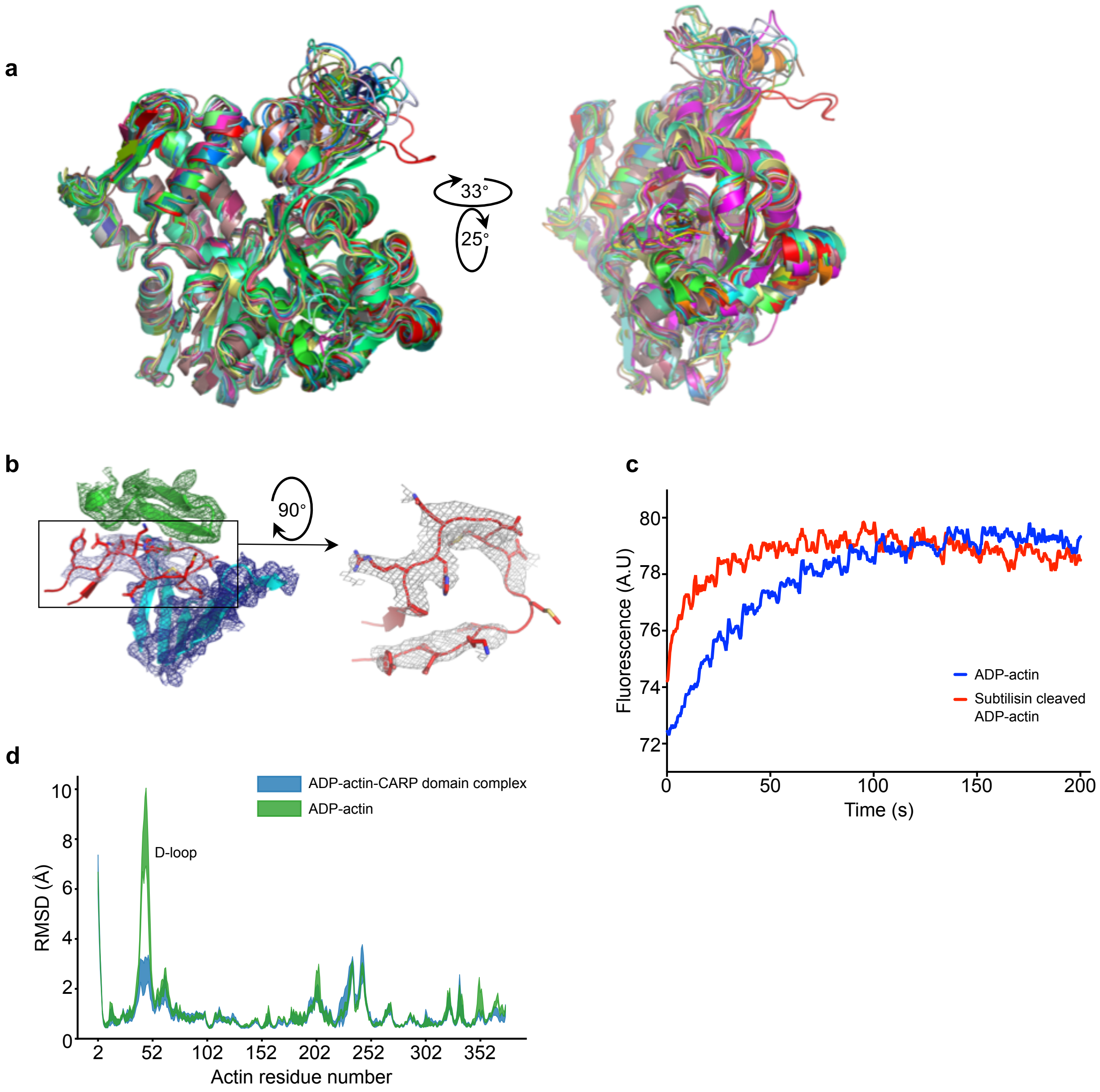

Supplementary Fig. 2. Effects of CARP domain on the conformation of the D-loop of actin. **(a)** Comparison of different D-loop conformations in 26 actin structures (Supplementary Table 2) where the D-loop is resolved (the CARP domain complexed with actin is depicted in red). **(b)** Electron density map of the D-loop conformation in the CARP-actin complex in our crystal structure ($2F_0$ - F_C, contoured at σ = 1.0). Actin molecule from the neighbouring asymmetric unit is shown in green and the CARP domain b-strand from interface 2 in teal. **(c)** A single experiment of nucleotide exchange of 0.5 µM subtilisin cleaved ADP-actin. Exchange of ADP to ε-ATP is ~3-fold faster than in untreated ADP-actin. **(d)** RMSD (root mean square deviation) per residue of the actin molecule in ADP-actin—CARP domain complex (System 1, Supplementary Table 3) and ADP-actin isolated from it (System 4, Supplementary Table 3). The calculations were performed over each 1.2 µs simulation separately. RMSD per residue with respect to the average actin structure was calculated after superposing the trajectories based on the actin alpha-carbons. Average RMSD per residue over the five trajectories and the standard deviation (band thickness in the plot) is reported.

Supplementary Figure 3

a

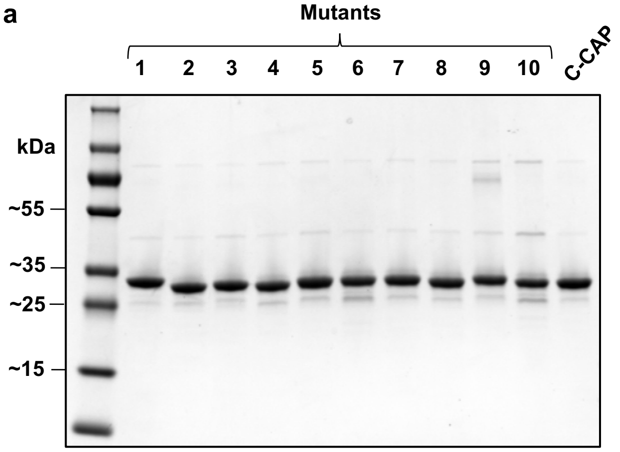

b

C-CAP protein	Retention volume (ml)	T_m (°C)
C-CAP	12.4	56
1	12.4	56
2	12.8	56
3	12.4	56
4	12.6	56
5	12.6	54
6	12.3	52
7	12.6	59
8	12.4	57
9	10.6	N.A*
10	12.2	49
	*weak signal	

c

#	Protein	K_d ADP-actin (μM)	K_d ATP-actin (μM)
	C-CAP$_{(217-474)}$	0.055	0.958
1	R253A L256A I260A	0.218	N.A
2	Δ^{266}ITHA^{269}	0.244	N.A
3	^{270}LKHV^{273} -> ^{270}AAAA^{273}	0.289	N.A
4	^{279}THKN^{282} -> ^{279}AAAA^{282}	0.087	4.94
5	K347A Y351A Y353A	> 1.0*	0.618
6	K365A N367A D372A	N.A**	0.881
7	D446A E449A	> 1.0*	0.887
8	Δ^{471}EIAG^{474}	0.009	0.801
9	Y418A F447A	N.A**	1.537
10	L339A Q399A D446A	0.345	0.934
	*estimated from raw data<br>**N.A = unmeasurable		

d

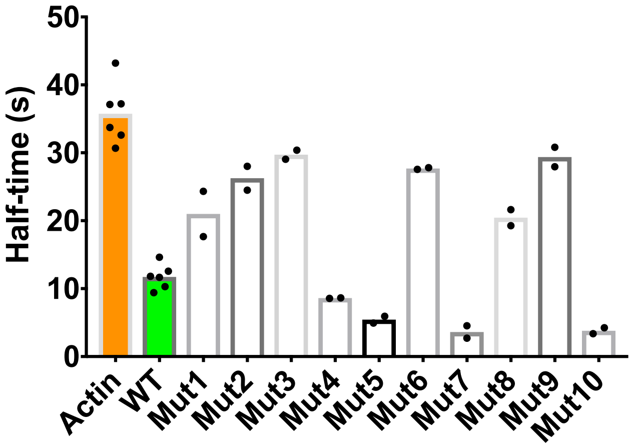

Supplementary Fig. 3. Biochemical properties of the C-CAP mutants. (a) SDS-PAGE analysis of the purified wild-type and mutant C-CAPs. **(b)** A table showing their corresponding elution volumes on gel filtration with SD200 Increase 10/300 from protein purification, and their representative unfolding temperatures measured (Tm) with Thermofluor. All mutant proteins, with the exception of mutant 9 (Y418A,F447A; indicated in red), were fully soluble and eluted in gel filtration at an identical volume compared to the wild-type C-CAP. Furthermore, thermal stability of these mutants confirmed that proteins are properly folded. **(c)** A summary of the affinities of C-CAP proteins for ADP- and ATP-G-actin from NBD-actin binding measurements. **(d)** Nucleotide exchange assay measuring the incorporation of 25 μM εATP to ADP-actin using 0.1 μM C-CAP proteins and 0.5 μM actin. In orange, actin nucleotide exchange rate in the absence of CAPs and in green the rate of nucleotide exchange with 0.1 μM wild-type (WT) C-CAP present. Each data point represents a value from an independent measurement. Half-times were calculated from the fit assuming one-phase association.

Supplementary Figure 4

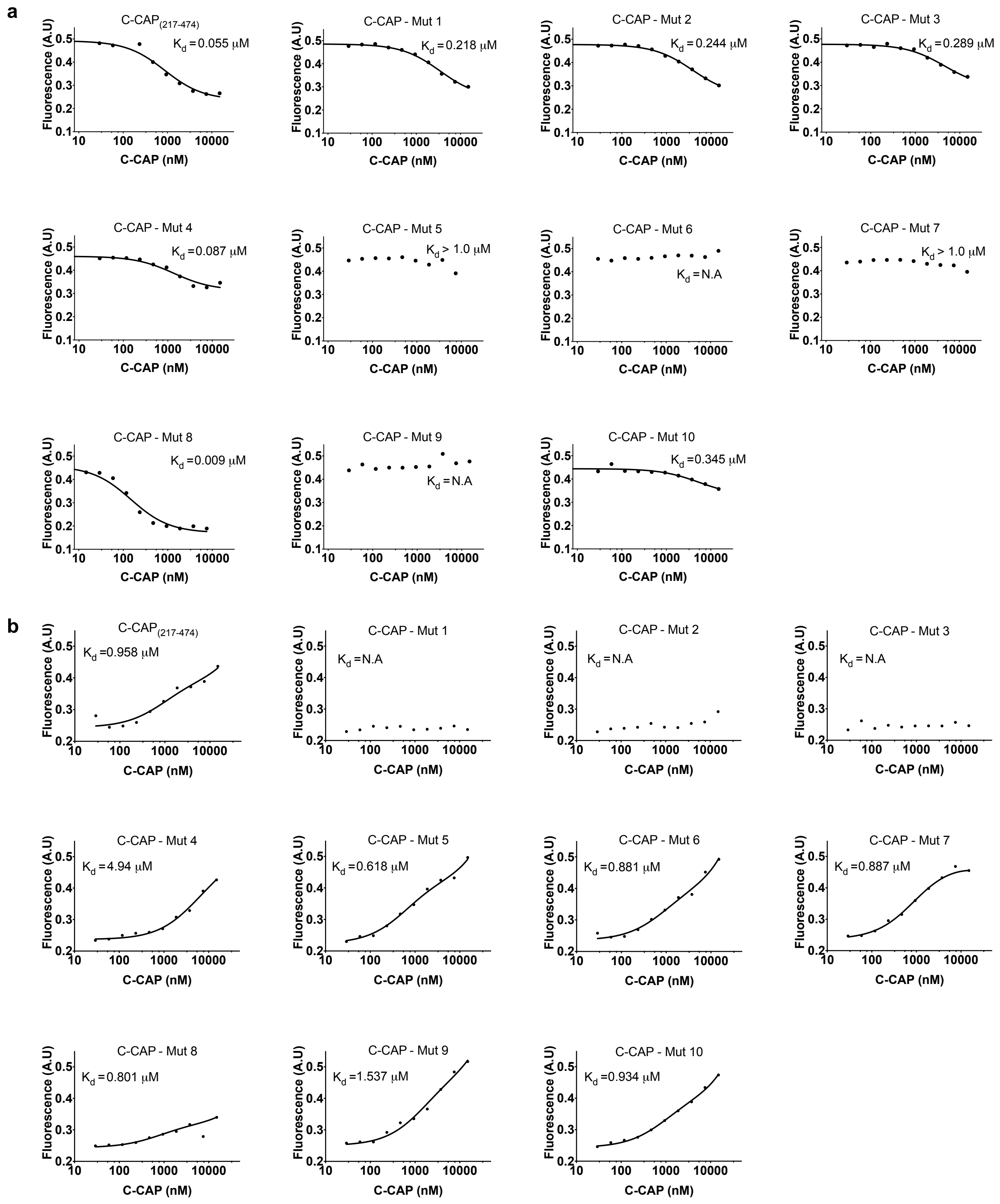

Supplementary Fig. 4. Representative raw binding data from the NBD-actin assays for wild-type and mutant C-CAPs. (a) NBD-assay with ADP-G-actin. Concentration of C-CAP was varied from 0 to 15 μM and the concentrations of actin and twinfilin$_{169\text{-}350}$ were 0.18 μM and 0.44 μM, respectively. Data were fitted in GraphPad Prism 7 assuming one-site competition with 0.03 μM affinity for twinfilin. Data are representative from a single titration experiment. (b) Direct NBD-assay for ATP-G-actin binding assuming one site binding in fit. C-CAP concentrations were varied from 0 to 15 μM, and the concentration of actin was 0.18 μM. Data are representative from a single titration experiment.

Supplementary Figure 5

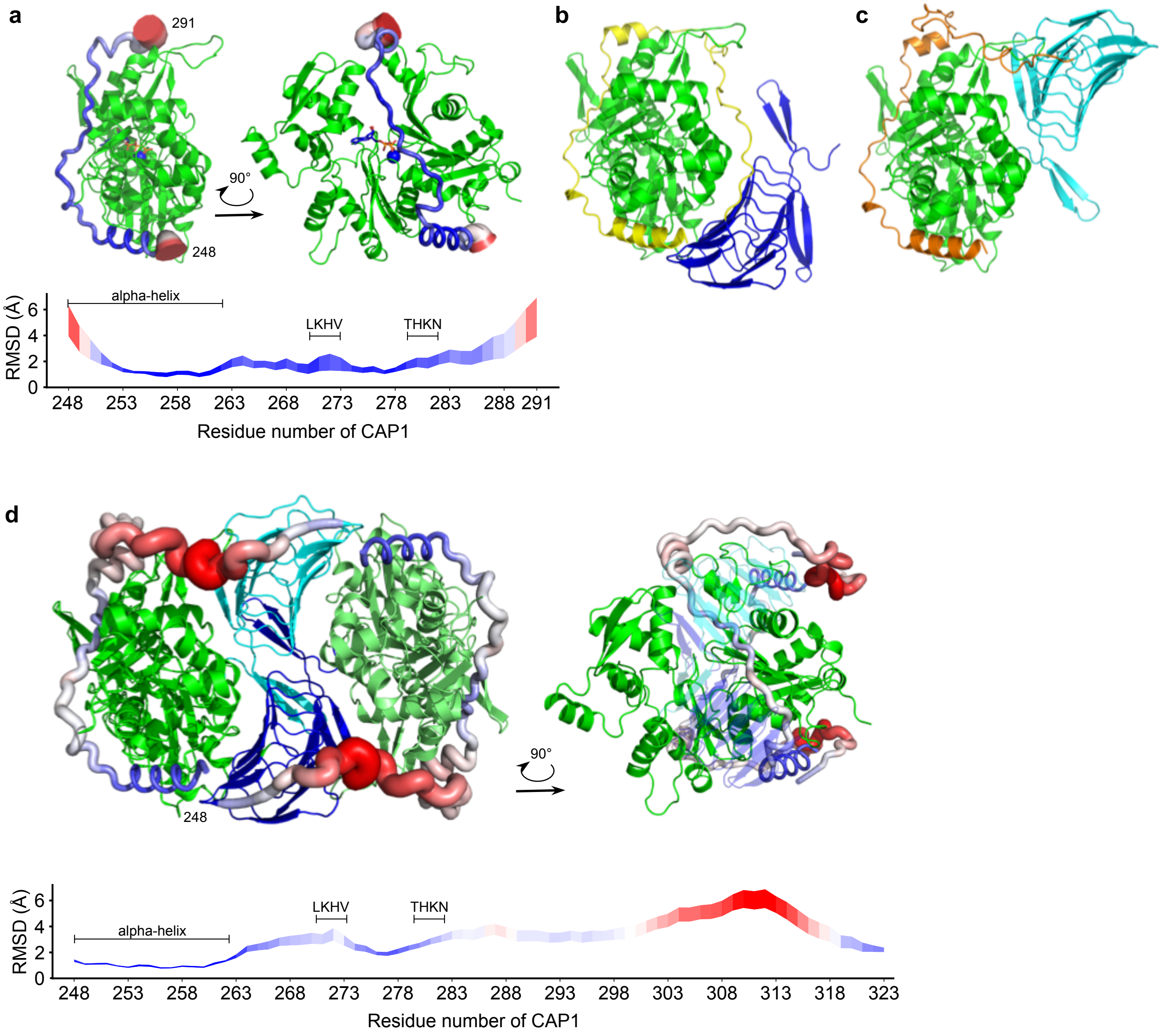

Supplementary Fig. 5. Molecular dynamics simulations of ADP-actin-CAP1$_{248\text{-}474}$ and ATP-actin-WH2 domain complexes. **(a)** Dynamics of the WH2 domain in the ATP-actin—WH2 domain complex (System 3, Supplementary Table 3). WH2 domain (residues 248-291) is shown in tube representation, where the tube thickness and the color correlate with the RMSD per residue. The calculations were performed over the last 200 ns of five 1.2 μs ATP-actin/WH2 domain simulations. RMSD per residue with respect to the average WH2 structure was calculated after superposing the trajectories based on the actin alpha-carbons. Average RMSD per residue over the trajectories and the standard deviation (band thickness in the plot) are reported. **(b-c)** Molecular modeling of the WH2 domain connection through PP2 to the CARP domain. The cis **(b)** and the trans **(c)** configurations of the WH2 - CARP domain connections are shown. **(d)** Dynamics of the WH2 domain and PP2 in the ADP-actin—CAP1$_{248\text{-}474}$ domain complex (System 2, Supplementary Table 3). The WH2 domain (residues 248-323) is shown in tube representation, where the tube thickness and the color correlate with the RMSD per residue. The calculations were performed over the last 200 ns of five 1.2 μs simulations. RMSD per residue with respect to the average WH2 structure was calculated after superposing the trajectories based on the actin alpha-carbons.

Supplementary Figure 6

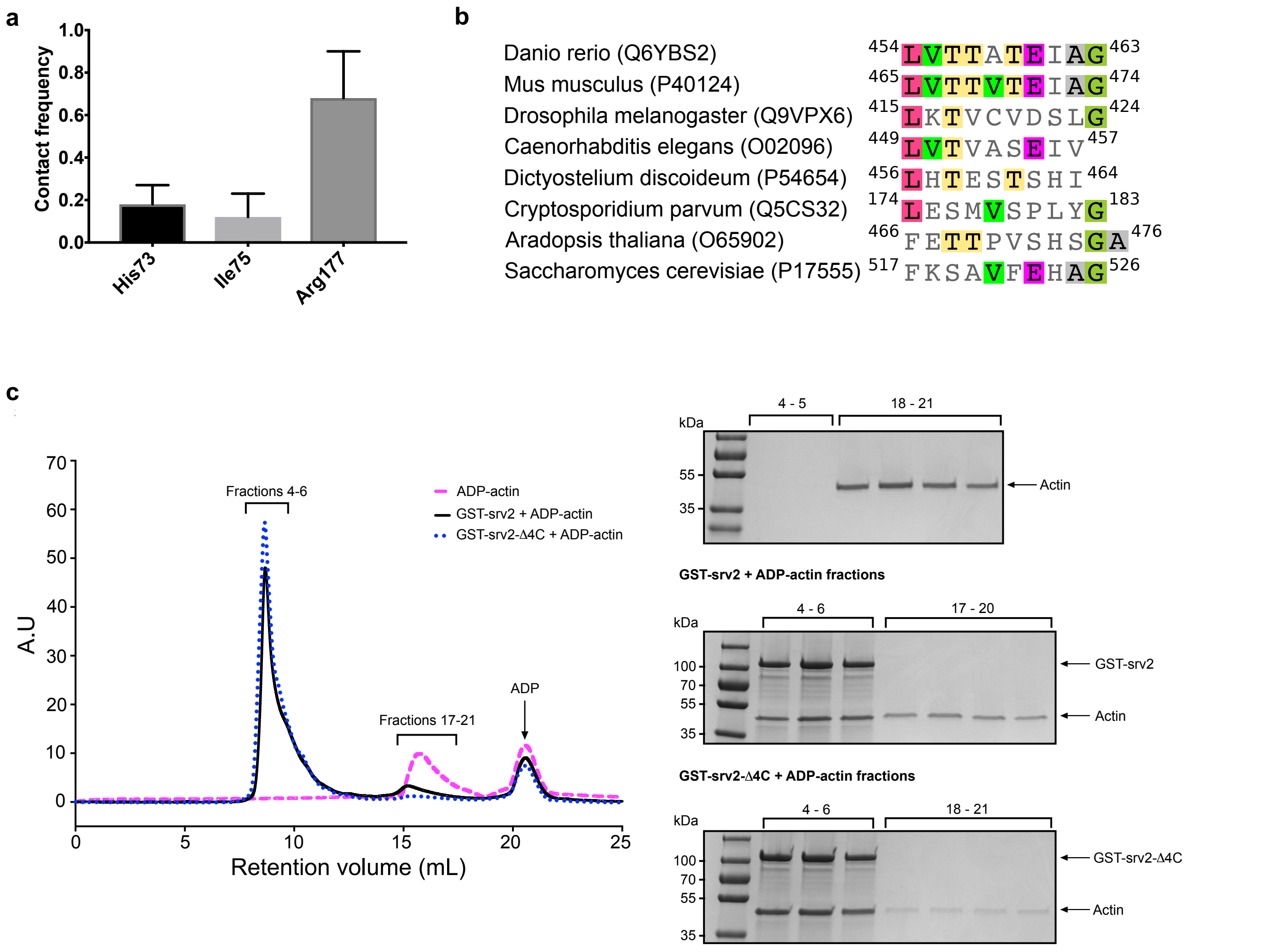

Supplementary Fig. 6. Interaction of the C-terminal tail of CAP with actin. (a) The contacts between the four C-terminal CARP domain residues and actin in ADP-actin—CARP domain complex simulations (System 1, Supplementary Table 3). Bar graph shows the average frequency of contacts (cutoff of 3.5 Å) that any CARP C-terminal residue (471-474) makes with actin residues over the five repeats; and error bars represent standard deviation of mean. See Fig. 4b, for the position of these residues in the nucleotide sensing region of actin. **(b)** Sequence alignment of the C-terminal regions of CAPs from representative organisms. Uniprot accession numbers are indicated in brackets. Different colors represent sequence conservation for the specific residues among aligned organisms. **(c)** Binding of GST-tagged full-length wild-type Srv2 and Srv2Δ4C mutant to ADP-actin monomers was analysed by size-exclusion chromatography. Retention of 300 µl of 10 µM of ADP-actin alone (in magenta), 10 µM of ADP-actin + 10 µM GST-Srv2 wild type (in black), and 10 µM of ADP-actin + 10 µM GST-Srv2-Δ4C (in blue) were analysed using Superdex 200 Increase 10/300 GL gel filtration column equilibrated in 5 mM HEPES, 100 mM NaCl, 0.05 mM ADP, 0.05 mM $MgCl_2$ and 0.5 mM β-mercaptoethanol. Elution profiles are on the left, and SDS-PAGE analysis of peak fractions is on the right. ADP-G-actin alone shows retention volume of ~ 15.7 ml. When GST-Srv2 polypeptides and ADP-actin are mixed together, ~ 70% of ADP-actin shifts to where GST-Srv2 elutes (~ 8.5 ml) and ~ 95% of ADP-actin to where GST-Srv2-Δ4C elutes (~ 8.5 ml).

Supplementary Figure 7

a

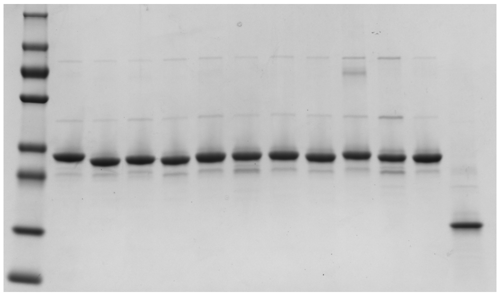

b

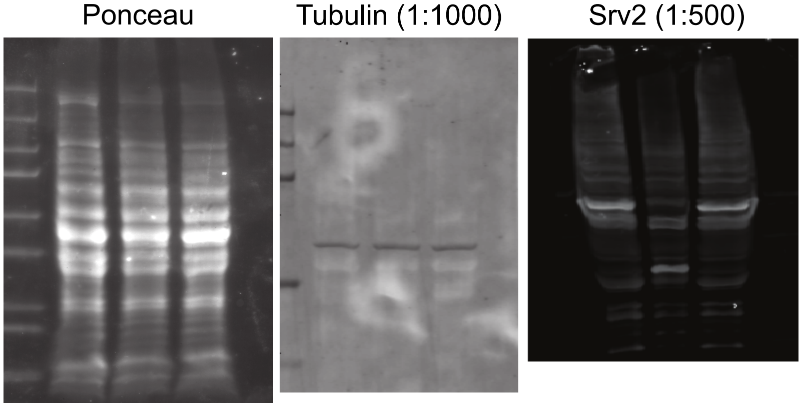

c

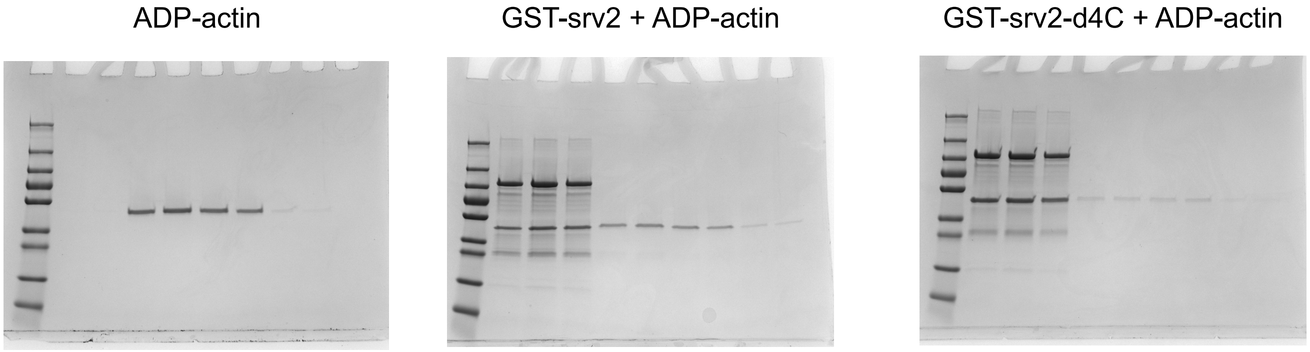

Supplementary Fig. 7. Uncropped images. (a) Raw image file for SDS-PAGE analysis of C-CAP mutants for Supplementary Figure 3a. **(b)** The raw image files for Western blot analysis of Figure 5a. **(c)** The raw image files for gel filtration fraction analysis from Supplementary Fig. 6c.

Supplementary Figure 8

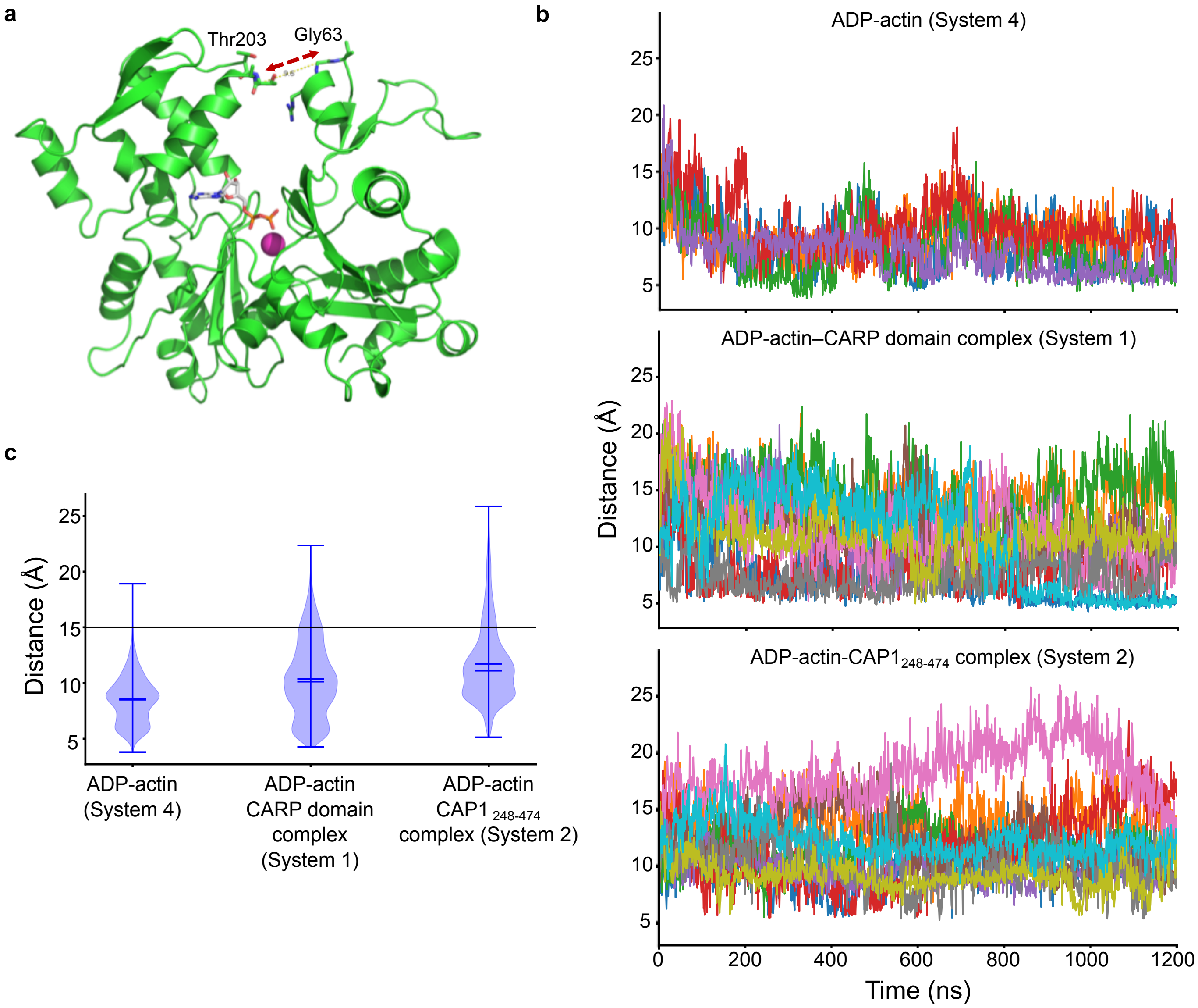

Supplementary Fig. 8. Analysis of the opening of the nucleotide-binding cleft. **(a)** Representation of the distances between Thr203 Cα in subdomain 4 and Gly63 Cα in subdomain 2 as a measure to examine opening of the nucleotide-binding cleft upon interaction with CAP. **(b)** Distances between Thr203 Cα and Gly63 Cα from molecular dynamics simulations of ADP-actin (System 4, Supplementary Table 3), ADP-actin—CARP complex (System 1, Supplementary Table 3), and ADP-actin—CAP1$_{248\text{-}474}$ (System 2, Supplementary Table 3) complexes. The distances are calculated for each actin in each simulation separately, resulting in 5 separate time traces for ADP-actin, and 10 for ADP-actin-CARP domain and ADP-actin-CAP1$_{248\text{-}474}$ complexes (shown in different colours). **(c)** Violin plots displaying the distribution of the distances, the means (lower lines), the medians (upper lines), as well as the minima and extrema over the last 1.0 μs of all simulations. The system was allowed to equilibrate for the first 200 ns.

Supplementary Table 1. Data collection and refinement statistics.

Data collection	*CARP-ADP-actin** (PDB 6fm2)
**Space group**	P 61 2 2
**Cell dimensions**	
a, b, c (Å)	73.83 73.83 453.37
α, β, γ (°)	90.00 90.00 120.00
**Resolution** (I / σI > 1.3)	39.57-2.8 Å (2.95-2.8 Å)
l axis ($CC_{1/2}$ > 0.3)	2.3 Å
hk plane ($CC_{1/2}$ > 0.3)	3.2 Å
**Number of unique reflections**	19109 (2749)
**Multiplicity**	6.0 (6.4)
**Completeness (%)**	98.2 (99.93)
R_{merge}	0.08 (1.371)
R_{pim}	0.035 (0.585)
I / σI	11.6 (1.3)
$CC_{1/2}$	0.999 (0.777)
Refinement	
R_{work} / R_{free} (%)	18.6/23.4 (23.7/33.8)
**Protein residues**	528
**No. atoms**	
Protein	4110
Ligand/ion	39
Water	79
**B-factors**	128.7
Wilson B-factor	85.8
Protein	129.5
Ligand/ion	102.1
Water	98.7
**Anisotropic B-factor** (B_{11}, B_{22}, B_{33})	-21.7511, -21.7511, 43.5022
**R.m.s. deviations**	
Bond lengths (Å)	0.010
Bond angles (°)	1.22
**Ramachandran plot**	
Outliers	2 (0%)
Allowed	38 (7%)
Favored	483 (93%)
**Clashscore (all-atoms)**	6

*highest resolution shell in brackets

Supplementary Table 2. The crystal structures of actin with D-loop modelled.

PDB ID (reference)	Name/Description	Actin state
3daw[7]	Structure of the ADF homology domain in complex with Actin	ATP
3w3d[9]	Crystal structure of smooth muscle G Actin DNase I complex	ATP
1atn[10]	Atomic structure of the Actin:DNAse I complex	ATP
1j6z[4]	Uncomplexed Actin	ADP
2a3z[11]	Ternary complex of the WH2 domain of WASP with Actin-DNAse I	ATP
4jhd[12]	Crystal structure of an Actin dimer in complex with the Actin nucleator Cordon-Bleu	ANP
4pkg[13]	Complex of ATP-Actin with the N-terminal Actin-binding domain of tropomodulin	ATP
4pki[13]	Complex of ATP-Actin with the C-terminal Actin-binding domain of tropomodulin	ATP
2a42[11]	Actin-DNAse I Complex	ATP
2zwh[14]	Model for the F-Actin structure	ADP
4rwt[12]c	Structure of Actin-Lmod complex	ANP
1h1v[15]	Structure of the G4-G6/Actin Complex	ATP
2btf[16]	The structure of crystalline profilin-β-Actin	ATP
3j8a[17]	F-Actin-Tropomyosin-EM structure	ADP
1hlu[18]	The structure of an open state of β-Actin at 2.65 A resolution	ATP
4cbw[19]	Crystal structure of Plasmodium berghei Actin I with D-loop from muscle Actin	ATP
4z94[20]	Actin Complex With a Chimera of Tropomodulin-1 and Leiomodin-1 Actin-Binding Site 2	ATP
3eks[21]	Crystal Structure of monomeric Actin bound to Cytochalasin D	ATP
3jbk[22]	Cryo-EM reconstruction of the metavinculin-Actin interface	ADP
2q97[23]	Complex of mammalian Actin with toxofilin from toxoplasma gondii	ATP
2a40[11]	Ternary complex of the WH2 domain of WAVE with Actin-DNAse I	ATP
2a41[11]	Ternary complex of the WH2 Domain of WIP with Actin-DNAse I	ATP
2d1k[24]	Ternary complex of the WH2 domain of MIM with Actin-DNAse I	ATP
2vcp[25]	Crystal structure of N-Wasp VC domain in complex with skeletal Actin	ATP
3ffk[26]	Crystal structure of human Gelsolin domains G1-G3 bound to Actin	ATP
3tu5[27]	Actin complex with Gelsolin segment 1 fused to Cobl segment	ATP
	26 structures in total	

Supplementary Table 3. Summary of the atomistic simulation systems.

System no.	System name	Notes on the initial protein configurations	No. of repeats		Simulation time (µs)	No. of waters	Box volume (nm^3)
**1**	ADP-actin—CARP domain complex	Crystal structure	5	×	1.2	59,000	1922
**2**	ADP-actin—$CAP1_{248\text{-}474}$	WH2 and PP2 domains are modeled in complex with System 1 in *trans* configuration.	5	×	1.2	59,000	1940
**3**	ATP-actin—WH2 domain complex	1) CARP and the PP2 domains are removed from System 2. 2) ADP is replaced with ATP.	5	×	1.2	25,000	809
**4**	ADP-actin	CARP is removed from System 1.	5	×	1.2	25,000	803

Supplementary Table 4. **Primer sequences used in this study.**

Plasmid	Explanation	Forward primer(s)	Reverse primer(s)
pPL973	pCoofy18-10xHIS-3C-mouse C-CAP(217-474) for biochemistry	CTGGAAGTTCTGTTCCAGGGGCCCAGTGGATTGCCATCTGGACCCTC	CCCCAGAACATCAGGTTAATGGCGCTATCCAGCGATTTCTGTCACTGTGG
pPL974	pCoofy18-10xHIS-3C-mouse CAP1(242-474) for crystallizations	CTGGAAGTTCTGTTCCAGGGGCCCACCAGTTCTGGTTCTGACGACTCTG	CCCCAGAACATCAGGTTAATGGCGCTATCCAGCGATTTCTGTCACTGTGG
pPL1064	pCoofy18-10xHIS-3C-mouse C-CAP(217-474)-mut1	GCTTCAGCAGCTTTTGCACAAGCAAATCAGGGGGAAAGCATCACACATGCCCTG	TGCTTGTGCAAAAGCTGCTGAAGCTGATGCAGAGTCGTCAGAACCAGAACTGGT
pPL1065	pCoofy18-10xHIS-3C-mouse C-CAP(217-474)-mut2	GGGGAAAGCCTGAAACATGTATCTGATGACATGAAGACTCACAAGAACC	CATGTTTCAGGCTTTCCCCCTGATTAATCTGTGCAAACAGTGCTGAG
pPL1066	pCoofy18-10xHIS-3C-mouse C-CAP(217-474)-mut3	GCAGCGGCAGCTGCATCTGATGACATGAAGACTCACAAGAACCCTGCCCTG	TGCAGCTGCCGCTGCATGTGTGATGCTTTCCCCCTGATTAATCTGTGC
pPL1067	pCoofy18-10xHIS-3C-mouse C-CAP(217-474)-mut4	CATGAAGGCTGCTGCAGCCCCTGCCCTGAAAGCTCAGAGCGG	GGCTGCAGCAGCCTTCATGTCATCAGATACATGTTTCAGGGCATGTGTGATGC
pPL1068	pCoofy18-10xHIS-3C-mouse C-CAP(217-474)-mut5	GCTCAAGTTGCTGCAATTGCAAAGTGTGTCAACACAACATTGCAAATCAAGGGC	TGCAATTGCAGCAACTTGAGCCAGCTCAGTGTCATCAATCACCAGGTTAGAAAC
pPL1069	pCoofy18-10xHIS-3C-mouse C-CAP(217-474)-mut6	GCAATTGCCTCCATTACAGTAGCTAACTGTAAGAAGCTTGGCCTGGTGTTTGATG	AGCTACTGTAATGGAGGCAATTGCGCCCTTGATTTGCAATGTTGTGTTGACACAC
pPL1071	pCoofy18-10xHIS-3C-mouse C-CAP(217-474)-mut7	GGCGGTGCTTTTAACGCGTTCCCAGTCCCCGAGCAGTTCAAGAC	GGAACGCGTTAAAAGCACCGCCTTCGGTAGGAATGAGGACATTCATCTCAG
pPL1072	pCoofy18-10xHIS-3C-mouse C-CAP(217-470)/mut8	CAGTGACATAGCGCCATTAACCTGATGTTCTGGGGAATATAAGCTTGC	GTTAATGGCGCTATGTCACTGTGGTGACCAACTTCTGTCCGTTC
pPL1073	pCoofy18-10xHIS-3C-mouse C-CAP(217-474)-mut9	**F447A:** AGGCGGTGATGCTAACGAGTTCCCAGTCCCCGAGCAG **Y418A:** GCCATGCTGCCCTGAGCAAGAACTCCCTGGACTGTGAGATAG	**F447A:** GGAACTCGTTAGCATCACCGCCTTCGGTAGGAATGAGGC **Y418A:** CTTGCTCAGGGCAGCATGGCAGCCATCTGTTTTGTTAATGG
pPL1075	pCoofy18-10xHIS-3C-mouse C-CAP(217-474)-mut10	**L339A:** GAGAATGTTTCTAACGCGGTGATTGATGACACTGAGCTGAAGCAGGTG **Q399A:** GATGTCAAAGTTGCGGTGATGGGAAAAGTGCCAACCATTTCCATTAAC **D446A:** AAGGCGGTCGCTTTAACGAGTTCCCAGTCCCCGAGC	**L339A:** CATCAATCACCGCGTTAGAAACATTCTCCTGGTTTTCCACTCTCC **Q399A:** TTTTCCCATCACCGCAACTTTGACATCCCTACTATTGATTATCTCCACAATG **D446A:** GGAACTCGTTAAAGCGACCGCCTTCGGTAGGAATGAGGACATTC

SUPPLEMENTARY REFERENCES

1. Krissinel, E. & Henrick, K. Inference of macromolecular assemblies from crystalline state. *J. Mol. Biol.* **372,** 774–97 (2007).
2. Hliscs, M. *et al.* Structure and function of a G-actin sequestering protein with a vital role in malaria oocyst development inside the mosquito vector. *J. Biol. Chem.* **285,** 11572–83 (2010).
3. Dodatko, T. *et al.* Crystal structure of the actin binding domain of the cyclase-associated protein. *Biochemistry* **43,** 10628–10641 (2004).
4. Otterbein, L. R., Graceffa, P. & Dominguez, R. The crystal structure of uncomplexed actin in the ADP state. *Science* **293,** 708–11 (2001).
5. Rould, M. A., Wan, Q., Joel, P. B., Lowey, S. & Trybus, K. M. Crystal Structures of Expressed Non-polymerizable Monomeric Actin in the ADP and ATP States. *J.Biol.Chem.* **281,** 31909–31919 (2006).
6. Laskowski, R. A. & Swindells, M. B. LigPlot+: multiple ligand-protein interaction diagrams for drug discovery. *J. Chem. Inf. Model.* **51,** 2778–86 (2011).
7. Paavilainen, V. O., Oksanen, E., Goldman, A. & Lappalainen, P. Structure of the actin-depolymerizing factor homology domain in complex with actin. *J. Cell Biol.* **182,** 51–9 (2008).
8. Porta, J. C. & Borgstahl, G. E. O. Structural basis for profilin-mediated actin nucleotide exchange. *J. Mol. Biol.* **418,** 103–16 (2012).
9. Sasaki, K., Sakabe, K., Sakabe, N., Kondo, H. & Shimomur, M. Refined structure and solvent network of chicken gizzard G-actin DNase 1 complex at 1.8A resolution. *Acta Crystallogr.,Sect.A* **49,** C111–C112 (1993).
10. Kabsch, W., Mannherz, H. G., Suck, D., Pai, E. F. & Holmes, K. C. Atomic structure of the actin:DNase I complex. *Nature* **347,** 37–44 (1990).
11. Chereau, D. *et al.* Actin-bound structures of Wiskott-Aldrich syndrome protein (WASP)-homology domain 2 and the implications for filament assembly. *Proc. Natl. Acad. Sci. U. S. A.* **102,** 16644–9 (2005).
12. Chen, X. *et al.* Structural basis of actin filament nucleation by tandem w domains. *Cell Rep* **3,** 1910–1920 (2013).
13. Rao, J. N., Madasu, Y. & Dominguez, R. Actin cytoskeleton. Mechanism of actin filament pointed-end capping by tropomodulin. *Science (80-.).* **345,** 463–467 (2014).
14. Oda, T., Iwasa, M., Aihara, T., Maeda, Y. & Narita, A. The nature of the globular- to fibrous-actin transition. *Nature* **457,** 441–445 (2009).
15. Choe, H. *et al.* The Calcium Activation of Gelsolin:Insights from the 3A Structure of the G4-G6/Actin Complex. *J.Mol.Biol.* **324,** 691 (2002).
16. Schutt, C. E., Myslik, J. C., Rozycki, M. D., Goonesekere, N. C. & Lindberg, U. The structure of crystalline profilin-beta-actin. *Nature* **365,** 810–816 (1993).
17. von der Ecken, J. *et al.* Structure of the F-actin--tropomyosin complex. *Nature* **519,** 114–117 (2015).
18. Chik, J. K., Lindberg, U. & Schutt, C. E. The structure of an open state of beta-actin at 2.65 A resolution. *J.Mol.Biol.* **263,** 607–623 (1996).
19. Vahokoski, J. *et al.* Structural Differences Explain Diverse Functions of Plasmodium Actins. *Plos Pathog.* **10,** 4091 (2014).
20. Boczkowska, M., Rebowski, G., Kremneva, E., Lappalainen, P. & Dominguez, R. How Leiomodin and Tropomodulin use a common fold for different actin assembly functions. *Nat Commun* **6,** 8314–8314 (2015).
21. Nair, U. B. *et al.* Crystal structures of monomeric actin bound to cytochalasin D. *J.Mol.Biol.* **384,** 848–864 (2008).
22. Kim, L. Y. *et al.* The Structural Basis of Actin Organization by Vinculin and Metavinculin. *J.Mol.Biol.* **428,** 10–25 (2016).
23. Lee, S. H., Hayes, D. B., Rebowski, G., Tardieux, I. & Dominguez, R. Toxofilin from Toxoplasma gondii forms a ternary complex with an antiparallel actin dimer. *Proc.Natl.Acad.Sci.Usa* **104,** 16122–16127 (2007).
24. Lee, S. H. *et al.* Structural basis for the actin-binding function of missing-in-metastasis. *Structure* **15,** 145–55 (2007).
25. Gaucher, J. F. *et al.* Interactions of isolated C-terminal fragments of neural Wiskott-Aldrich syndrome protein (N-WASP) with actin and Arp2/3 complex. *J. Biol. Chem.* **287,** 34646–34659 (2012).
26. Nag, S. *et al.* Ca2+ binding by domain 2 plays a critical role in the activation and stabilization of gelsolin. *Proc.Natl.Acad.Sci.USA* **106,** 13713–13718 (2009).
27. Durer, Z. A. *et al.* Structural States and dynamics of the d-loop in actin. *Biophys J.* **103,** 930–939 (2012).
