# Supplementary material for: Structural basis of actin monomer re-charging by cyclase-associated protein
Source: Nat Commun. 2018 May 14;9:1892. doi: 10.1038/s41467-018-04231-7 (PMC5951797; doi:10.1038/s41467-018-04231-7)
Supplement: Supplementary file 2 — Peer Review Report [file 41467_2018_4231_MOESM2_ESM.pdf]

Reviewers' comments:

Reviewer #1 (Remarks to the Author):

In their study “Structural basis of actin monomer re-charging by cyclase-associated protein” the authors provide a thorough description of the molecular mechanism by which the WH2 and CARP domains of cyclase-associated protein associate with actin monomers, how this leads to an acceleration of nucleotide exchange, and finally re-charging of actin monomers with ATP. The presented experimental results are original and of the highest quality. This outstanding contribution to the scientific literature will certainly influence further research in the cell motility field and is of interest for the wider scientific community.
My only suggestion for a change is a clarification in the introduction. Here, the authors should mention that two CAP proteins (CAP1 and CAP2) are produced in mammalian cells (yeast only has one CAP protein, which is related to CAP1) and that their work addresses the function of CAP1.

Reviewer #2 (Remarks to the Author):

This study determines structure of CAP1 bound to actin. The binding is uniquely on the back surface of actin. The authors go on to perform some elegant functional studies to test some of the predictions, particularly about the role of the c-terminus in ATP exchange. This is novel and elegant study of a first-in-class nucleotide exchanger for actin.

1 183 and fig 5 There is no mention of mut 6 in the paragraph beginning on line 183. This paragraph should be rewritten to clarify the point about heterodimers. Also, mixing heterodimers with actin at the appropriate ratios does not necessarily demonstrate actin binding as predicted. There may be ADP actin binding by mut 6 as a heterodimer that is not seen as a homodimer. Likewise, mutant 6, delta4C - wild-type heterodimers may not bind to actin. The manuscript would be tighter if it downplayed this figure, perhaps sending it to supplemental data or developing it into another study on dimerization. This would focus the paper on the points in the abstract.

2 Figure 6. The mutant has phenotypes similar to the loss of the entire carp domain. Confirming that the delta4C mutant in the yeast protein does not affect actin binding is central to the evidence for their claim that the deletion mutant affects only ADP exchange. Actin binding etc. was extensively documented with the truncation constructs isolated from E. Coli. However, actin binding was not tested in yeast. The reexpression construct has the OD and the HFD domains which may indirectly affect actin binding. Additionally, the actin binding domain is close to the deleted residues and may be damaged or misfolded when expressed in yeast. It is important to confirm actin monomer binding by the mutants expressed in yeast through immunoprecipitations and/or size exclusion analysis.

3 I would like to see some discussion of the ADP exchange mechanism compared to the GEF-GDP exchange mechanism by SOS on RAS. There are some similarities in that part of the GEF inserts into the nucleotide binding pocket to dislodge the nucleotide and that an unstructured loop on Ras switch 2 (or in this case actin) is stabilized by the association (Boriack-Sjodin, 1998).

4
minor
1 Arp2/3 and line 52. The topic of the paper is about treadmilling. Nucleation is an additional step not mentioned in the 4 step model of treadmilling. Either drop discussion of nucleation (and perhaps arp 2/3) or add nucleation to the steps of treadmilling.

Line 121 typo: displays also other

Reviewer #3 (Remarks to the Author):

The work provides an interesting account of the investigation of interactions between actin and cyclase-associated protein, using an integrative approach, in which the different techniques are used in a proper, complementary fashion. Focusing on the molecular dynamics simulations, I can attest that the strategy for constructing the starting structures, based upon directly and indirectly available information, and with sufficient assessment of the validity of the necessary assumptions, is in line with the state of the art. The number of simulations, choice of force field and the length of the simulations is suitable for the questions addressed. What I particularly like is how the results of the simulations are coupled back to the structural and biochemical data and vice versa, taking care not to overinterpret. I have no particular criticism regarding the work as is. The only direct question that comes up, and which the authors might care to comment on briefly, is what the difference is between ATP and ADP loaded actin with respect to the interactions.

Summary of Revisions

Reviewer #1 (Remarks to the Author):

In their study "Structural basis of actin monomer re-charging by cyclase-associated protein" the authors provide a thorough description of the molecular mechanism by which the WH2 and CARP domains of cyclase-associated protein associate with actin monomers, how this leads to an acceleration of nucleotide exchange, and finally re-charging of actin monomers with ATP. The presented experimental results are original and of the highest quality. This outstanding contribution to the scientific literature will certainly influence further research in the cell motility field and is of interest for the wider scientific community.

We thank the reviewer for positive comments.

My only suggestion for a change is a clarification in the introduction. Here, the authors should mention that two CAP proteins (CAP1 and CAP2) are produced in mammalian cells (yeast only has one CAP protein, which is related to CAP1) and that their work addresses the function of CAP1.

We have now added sentences to the 'Introduction' (lines 66-68) and 'Results' (lines 88-89) stating that two CAP isoforms exist in mammalian cells, and that our structural work focused on the non-muscle CAP1 isoform.

Reviewer #2 (Remarks to the Author):

This study determines structure of CAP1 bound to actin. The binding is uniquely on the back surface of actin. The authors go on to perform some elegant functional studies to test some of the predictions, particularly about the role of the c-terminus in ATP exchange. This is novel and elegant study of a first-in-class nucleotide exchanger for actin.

We thank the reviewer for the positive feedback and excellent suggestions.

1) 183 and fig 5 There is no mention of mut 6 in the paragraph beginning on line 183. This paragraph should be rewritten to clarify the point about heterodimers. Also, mixing heterodimers with actin at the appropriate ratios does not necessarily demonstrate actin binding as predicted. There may be ADP actin binding by mut 6 as a heterodimer that is not seen as a homodimer. Likewise, mutant 6, delta4C - wild-type heterodimers may not bind to actin. The manuscript would be tighter if it downplayed this figure, perhaps sending it to supplemental data or developing it into another study on dimerization. This would focus the paper on the points in the abstract.

We agree with the reviewer that, without obtaining specific actin-binding data on the heterodimeric constructs, our functional data is somewhat preliminary. As suggested, this figure was omitted from the revised version of the manuscript.

2) Figure 6. The mutant has phenotypes similar to the loss of the entire carp domain. Confirming that the delta4C mutant in the yeast protein does not affect actin binding is central to the evidence for their claim that the deletion mutant affects only ADP exchange. Actin binding etc. was extensively documented with the truncation constructs isolated from E. Coli. However, actin binding was not tested in yeast. The reexpression construct has the OD and the HFD domains which may indirectly affect actin binding. Additionally, the actin binding domain is close to the deleted residues and may be damaged or misfolded when expressed in yeast. It is important to

confirm actin monomer binding by the mutants expressed in yeast through immunoprecipitations and/or size exclusion analysis.

We performed the suggested coIP experiment by using extracts from wild-type and *srv2-ΔC4* yeast cells. Unfortunately, our available Srv2 antibodies are against the actin-binding CARP domain of the protein and hence they compete with actin monomer-binding. Thus, we did not succeed in co-precipitating actin from yeast extracts using these antibodies. Moreover, gel-filtration experiments on yeast extracts would require latrunculin A (to keep actin in monomeric form), and this is likely to interfere with the CAP-actin interaction. Therefore, we applied an alternative strategy to confirm that the Δ4C mutant does not interfere with protein folding or actin monomer binding in the context of full-length protein. We expressed GST-tagged full-length Srv2 and Srv2Δ4C in *E. coli,* purified the proteins, and examined their ADP-G-actin binding activities by a gel filtration experiment. The new data (shown in supplementary Fig. S6c and discussed on lines 171-172) demonstrate that both full-length Srv2 and Srv2Δ4C bind ADP-G-actin with high affinity. Consistent with the data on C-terminal halves of Srv2 (Fig. 4f), the full-length Srv2Δ4C appears to bind ADP-G-actin with slightly higher affinity compared to wild-type Srv2. Moreover, our gel filtration experiments provided evidence that both full-length Srv2 and Srv2Δ4C form similar large oligomers.

We would also like to point out that earlier studies (i.e. Chaudhry *et al*. 2014) demonstrated that the N-terminal and C-terminal halves of Srv2/CAP function independently of each other both *in vivo* and *in vitro*, and that based on the Western blot analysis (Fig. 5a), the levels Srv2 in wild-type and *srv2-Δ4C* strain are identical indicating that the protein is correctly folded in yeast cells. We did also consider integrating epitope tags at the C-termini of Srv2 and Srv2Δ4C to perform pull-downs from yeast extracts and thereby further demonstrate that the full-length proteins fold correctly and bind actin. However, we did not want to position tags so close to the active C-terminal site that is critical for nucleotide exchange, i.e., where the Δ4C mutation lies, and integrated N-terminal tags are problematic, as they disrupt the promoter region.

3) I would like to see some discussion of the ADP exchange mechanism compared to the GEF-GDP exchange mechanism by SOS on RAS. There are some similarities in that part of the GEF inserts into the nucleotide binding pocket to dislodge the nucleotide and that an unstructured loop on Ras switch 2 (or in this case actin) is stabilized by the association (Boriack-Sjodin, 1998).

This is an interesting point. We have now included discussion on the differences between the nucleotide exchange mechanisms of cyclase-associated protein and GEFs (lines 224-226).

4) Arp2/3 and line 52. The topic of the paper is about treadmilling. Nucleation is an additional step not mentioned in the 4 step model of treadmilling. Either drop discussion of nucleation (and perhaps arp 2/3) or add nucleation to the steps of treadmilling.

Thank you for pointing this out. We have now added nucleation as the first step for treadmilling (line 45).

Line 121 typo: displays also other

We have corrected this spelling mistake.

Reviewer #3 (Remarks to the Author):

The work provides an interesting account of the investigation of interactions between actin and cyclase-associated protein, using an integrative approach, in which the different techniques are used in a proper, complementary fashion. Focusing on the molecular dynamics simulations, I can attest that the strategy for constructing the starting structures, based upon directly and indirectly available information, and with sufficient assessment of the validity of the necessary assumptions, is in line with the state of the art. The number of simulations, choice of force field and the length of the simulations is suitable for the questions addressed. What I particularly like is how the results of the simulations are coupled back to the structural and biochemical data and vice versa, taking care not to overinterpret. I have no particular criticism regarding the work as is. The only direct question that comes up, and which the authors might care to comment on briefly, is what the difference is between ATP and ADP loaded actin with respect to the interactions.

This is an interesting point. Our mutagenesis experiments show that, whereas the CARP domain is critical for ADP-G-actin binding, it does not contribute to interactions with ATP-actin. In the course of this study, we also performed simulations of the CARP domain ATP-G-actin complex, but these did not reveal a clear explanation for the actin nucleotide-state specificity of the CARP domain. Several recent studies have provided evidence that ADP- and ATP-actin molecules are structurally very similar to each other, and the main difference between ATP- and ADP-actin may thus be linked to their internal dynamics. We agree that in the future it will be very important to uncover why most actin-binding proteins (including cyclase-associated protein) preferentially interact with either ATP- or ADP-actin, but we feel that this is beyond the scope of the present study. This is now mentioned in the 'Discussion' (lines 206-208).

REVIEWERS' COMMENTS:

Reviewer #2 (Remarks to the Author):

This is an impressive contribution to the field and the authors addressed most points adequately with additional data.

However, there is one point in the introduction that is still problematic and needs to be rephrased to accurately describe the differences between actin dynamics, nucleation and treadmilling. Lines 45 to 53 still confuse treadmilling and nucleation. Nucleation is distinct from treadmilling and is accelerated by ARP 2/3 and formins. Nucleation, formins and ARP 2/3 should not be part of their 5 step model of treadmilling. Their 5 step model accurately describes "actin dynamics", but just steps 2-5 describe treadmilling. Treadmilling proceeds without nucleation; an aged filament does not need nucleation to treadmill. This is clearly not the place for long winded definitions of these three concepts, but some rephrasing is required to accurately describe treadmilling.

Summary of Revisions

Reviewer #2 (Remarks to the Author):

However, there is one point in the introduction that is still problematic and needs to be rephrased to accurately describe the differences between actin dynamics, nucleation and treadmilling. Lines 45 to 53 still confuse treadmilling and nucleation. Nucleation is distinct from treadmilling and is accelerated by ARP 2/3 and formins. Nucleation, formins and ARP 2/3 should not be part of their 5 step model of treadmilling. Their 5 step model accurately describes "actin dynamics", but just steps 2-5 describe treadmilling. Treadmilling proceeds without nucleation; an aged filament does not need nucleation to treadmill. This is clearly not the place for long winded definitions of these three concepts, but some rephrasing is required to accurately describe treadmilling.

We have now edited the 'Introduction' to specify that actin filament nucleation and treadmilling are two separate processes that both are needed for actin-based processes in cells.
